# Integrated transcriptome and methylome analysis in youth at high risk for bipolar disorder: a preliminary analysis

**DOI:** 10.1038/tp.2017.32

**Published:** 2017-03-14

**Authors:** G R Fries, J Quevedo, C P Zeni, I F Kazimi, G Zunta-Soares, D E Spiker, C L Bowden, C Walss-Bass, J C Soares

**Affiliations:** 1 Translational Psychiatry Program, Department of Psychiatry and Behavioral Sciences, McGovern Medical School, University of Texas Health Science Center at Houston (UTHealth), Houston, TX, USA; 2 Center of Excellence on Mood Disorders, Department of Psychiatry and Behavioral Sciences, McGovern Medical School, The University of Texas Health Science Center at Houston (UTHealth), Houston, TX, USA; 3 Neuroscience Graduate Program, The University of Texas Graduate School of Biomedical Sciences at Houston, Houston, TX, USA; 4 Laboratory of Neurosciences, Graduate Program in Health Sciences, Health Sciences Unit, University of Southern Santa Catarina (UNESC), Criciúma, SC, Brazil; 5 Department of Psychiatry, University of Texas Health Science Center at San Antonio, San Antonio, TX, USA

## Abstract

First-degree relatives of patients with bipolar disorder (BD), particularly their offspring, have a higher risk of developing BD and other mental illnesses than the general population. However, the biological mechanisms underlying this increased risk are still unknown, particularly because most of the studies so far have been conducted in chronically ill adults and not in unaffected youth at high risk. In this preliminary study we analyzed genome-wide expression and methylation levels in peripheral blood mononuclear cells from children and adolescents from three matched groups: BD patients, unaffected offspring of bipolar parents (high risk) and controls (low risk). By integrating gene expression and DNA methylation and comparing the lists of differentially expressed genes and differentially methylated probes between groups, we were able to identify 43 risk genes that discriminate patients and high-risk youth from controls. Pathway analysis showed an enrichment of the glucocorticoid receptor (GR) pathway with the genes *MED1*, *HSPA1L*, *GTF2A1* and *TAF15*, which might underlie the previously reported role of stress response in the risk for BD in vulnerable populations. Cell-based assays indicate a GR hyporesponsiveness in cells from adult BD patients compared to controls and suggest that these GR-related genes can be modulated by DNA methylation, which poses the theoretical possibility of manipulating their expression as a means to counteract the familial risk presented by those subjects. Although preliminary, our results suggest the utility of peripheral measures in the identification of biomarkers of risk in high-risk populations and further emphasize the potential role of stress and DNA methylation in the risk for BD in youth.

## Introduction

Bipolar disorder (BD) is a devastating mental disorder with a prevalence of 1–2% and increased rates of several chronic comorbid medical conditions.^1^ Even though BD is highly heritable, most studies on its genetic basis have been conducted with chronically ill adults and therefore it is not known whether abnormalities found in patients precede the onset of illness, emerge during early illness development or follow BD onset. Particularly, the role that genes play in triggering onset of BD is not clear, but is believed to involve the interaction between many susceptibility genes of small effect and a broad range of environmental factors.^2^

First-degree relatives of BD patients are at increased risk for BD and other severe mental illnesses, and present a higher polygenic load of risk variants than control individuals.^3^ Particularly, offspring of bipolar parents present a fourfold increased risk of developing BD compared to offspring of healthy parents.^4^ Therefore, the search for biomarkers of risk in these individuals is urged and would allow an opportunity for prevention or early intervention.

DNA methylation, being modulated by both genetic background^5^ and environmental exposure,^6^ may be a marker of risk/resilience and a trigger for the development of BD.^7^ Further, DNA methylation can change the expression of the key genes, potentially contributing to disease susceptibility. Previous studies have suggested that the combined assessment of gene expression and methylation data outperforms either data modality in identifying disease susceptibility *loci*, even in relatively small sample sizes.^8^ Of note, the study of such markers in peripheral blood cells is warranted, given the easy access to tissue allowing for longitudinal comparisons in high-risk subjects. In addition, peripheral pathways, such as inflammatory and metabolic processes, have been consistently associated with BD and its risk,^9^ and blood DNA methylation has been shown to correlate with brain volume^10^ and symptoms of major psychiatric disorders, including depression^11^ and BD.^12^

We hypothesized that DNA methylation and gene expression events in peripheral pathways would discriminate between unaffected subjects at high risk for BD and healthy controls and could be promising biomarkers to monitor early presentation and prodromal symptoms in youth at risk for developing BD, because of the presence of a parent with a diagnosis of BD. Specifically, we aimed to integrate gene expression and DNA methylation data to identify risk genes of biological and functional relevance for the risk of BD in youth.

## Materials and methods

### Sample

This study protocol was approved by the local institutional review board, and informed consent was obtained from all the participants and parents/guardians. We selected 18 children and adolescents from three groups: patients diagnosed with pediatric BD (*n*=6), unaffected offspring of a BD type I parent (*n*=6; high risk) and healthy controls without any family history of psychiatric disorders in a first-degree relative (*n*=6; low risk) matched for age, gender, race, ethnicity and pubertal development (Table 1). Subjects were recruited as part of the Houston Area Pediatric Bipolar Registry. The Kiddie-Sads-Present and Lifetime Version^13^ was utilized to confirm or rule out DSM-IV Axis I disorders among the youth, which was confirmed subsequently in a clinical evaluation with a research psychiatrist. The affective state was assessed with the Children's Depression Rating Scale and the Young Mania Rating Scale.^14, 15^ Functioning was assessed by the Global Assessment of Functioning scale. To assess environment, family functioning ratings were assessed using the parent-rated scale Family Environment Scale,^16^ which provides information about family strength and problem areas divided into three dimensions (family relationship, personal growth and system maintenance). These are assessed in 10 subscales, of which cohesion and conflict in the family relationship domain have been the most consistently altered factors in family functioning of BD.^17, 18, 19^

For the BD offspring group, at least one biological parent met the DSM-IV criteria for BD type I, as determined by the Structured Clinical Interview for the DSM-IV Axis I Disorders interview. These high-risk subjects did not show any affective or non-affective diagnoses at the time of enrollment, including anxiety or externalizing problems, both of which have been shown to predict the prospective development of an affective disorder in this vulnerable population.^20^ Exclusion criteria for all participants included: (a) current major medical problems; (b) previous history of neurologic disorders, including head injury with loss of consciousness; (c) pregnancy; and (d) family history of a hereditary neurological disorder. High-risk offspring were recruited as their BD parent presented for evaluation at the inpatient or outpatient programs of the UT Center of Excellence on Mood Disorders.

### Transcriptome profiling

Heparin-anticoagulated blood from each fasting subject was used for the isolation of peripheral blood mononuclear cells (PBMCs) using the Ficoll-Paque (GE Healthcare, Little Chalfont, UK) density gradient centrifugation protocol, followed by isolation of RNA with the RNease Plus Mini kit (Qiagen, Hilden, Germany). After quantification on NanoDrop (Thermo, Waltham, MA, USA), the integrity of RNA samples was assessed by the Agilent RNA 6000 Nano Kit (Agilent Technologies, Santa Clara, CA, USA) on a Bioanalyzer (Agilent Technologies), and samples (RNA integrity number >9.8) were subsequently labelled into biotinylated cRNAs with the TargetAmp-Nano Labeling Kit (Epicentre, Madison, WI, USA). Genome-wide expression levels were measured using the Human HT-12 v4 Expression BeadChip Array (Illumina, San Diego, CA, USA) according to the manufacturer's instructions and scanned on an iScan Microarray Scanner (Illumina). Gene expression data were later quantile-normalized and exported into text files using GenomeStudio software v2011.1 (Illumina), and data analyses were performed using JMP Genomic (version 6.0, SAS Institute, Cary, NC, USA). Student's *t*-test was used in JMP to analyze values, with a Benjamini–Hochberg false discovery rate correlation of 1%, *α*=0.05, and a cutoff of –log_10_ (*P*-value) >1.5. A few genes shown to be differentially expressed between groups were selected for validation by quantitative real-time PCR, as described in the Supplementary Material.

### Methylome profiling

DNA (600 ng) was isolated from PBMCs using the DNeasy Blood and Tissue Kit (Qiagen) and bisulfite-converted with the EZ DNA Methylation Kit (Zymo Research, Irvine, CA, USA). Methylation levels were interrogated using the Infinium HumanMethylation450 BeadChip array (Illumina), according to the manufacturer's instructions, and analyzed on GenomeStudio v2011.1. Details of the analysis are described in the Supplementary Material. For the identification of biologically relevant DNA methylation differences, differentially methylated probes (DMPs) were selected based on a differential *P*-value<0.01 and an absolute delta beta value (magnitude of the difference between groups)⩾0.3. Correlation between the average signal for the gene expression and the beta value for each gene was performed using GenomeStudio v2011.1.

### Identification of risk genes and pathway analysis

‘Risk genes' were identified as those genes that were concordant in the lists of differentially expressed genes or DMPs between controls versus high-risk offspring and controls versus BD patients. These genes were then uploaded into Ingenuity Pathway Analysis (IPA, Qiagen) for the assessment of enrichments in canonical pathways and networks. This was followed by literature and database mining to check for evidence of associations between the risk genes identified in our analysis and previous studies in BD patients (described in the Supplementary Material).

### Treatment of lymphoblastoid cell lines and cell-based assays

Lymphoblastoid cell lines from 10 healthy controls and 10 unrelated adult BD-I patients matched for age, gender, ethnicity and race (Table 1) were generated from leucocytes using LeucoPREP brand cell separation tubes (Becton Dickinson Labware, Bedford, MA, USA) and transformed using Epstein–Barr virus. Cells were initially counted and seeded onto 12-well plates (0.25 × 10^6^ cells per well) in RPMI 1640 medium (Sigma-Aldrich, St Louis, MO, USA) containing 15% fetal bovine serum (Gibco, Carlsbad, CA, USA) and 1% penicillin-streptomycin (Gibco), after which they were treated with either 1 or 5 μM 5-aza-2'-deoxycytidine (5AzadC; Sigma-Aldrich) for 96 h with no changing of medium.^21^ This treatment condition has been previously shown to significantly reduce global DNA methylation in lymphoblastoid cell lines.^21^ Cell viability was assessed by the (3-(4,5-dimethylthiazol-2-yl)-2,5-diphenyltetrazolium bromide) tetrazolium (MTT) reduction assay.^22^ Treatment with 5AzadC was followed by RNA and DNA isolation with the RNeasy Plus Mini kit (Qiagen) and DNeasy Blood & Tissue Kit (Qiagen), respectively. Reduction in the levels of 5-methylcytosine (5-mC) was confirmed with the 5-mC DNA ELISA Kit (Zymo Research), according to the manufacturer's instructions, and real-time quantitative PCR was later performed to assess the levels of *MED1*, *GTF2A1*, *HSPA1L* and *TAF15* (Supplementary Material).

### Glucocorticoid receptor response in lymphoblastoid cell lines

Lymphoblastoid cells (same as above) were treated with or without 10^−7^ M dexamethasone (Sigma-Aldrich) for 4 h (acute treatment) or 48 h (chronic treatment) after overnight stabilization in a medium containing charcoal-treated fetal bovine serum (Gemini Bio, West Sacramento, CA, USA). Cell viability was assessed by the MTT reduction assay.^22^ Immediately after treatment, cells were collected and subjected to RNA isolation using the RNeasy Plus Mini Kit (Qiagen), according to the manufacturer's instructions. The expression levels of three different glucocorticoid receptor (GR)-responsive genes (*FKBP5*, *TSC22D3* and *PER1*) were measured by real-time quantitative PCR (Supplementary Material).

### Statistical analysis

Data analyses were carried out using IBM SPSS Statistics 23 (IBM, Armonk, NY, USA). Descriptive statistics were used to report demographic and clinical characteristics of the sample. Normality of data distribution was assessed with Shapiro–Wilk's test and histogram visualization. Categorical data were compared with chi-square tests or Fisher's exact tests. One-way analysis of variance was performed to compare parametric continuous demographic variables between groups. Independent Student's *t*-test was used to compare age between the adult patients and controls used in the lymphoblastoid cell line experiments. Factorial analysis of variance was used to analyze gene expression levels after treatment of cells with 5AzadC or dexamethasone. Analysis of covariance was also used to compare gene expression (*MED1*, *GTF2A1*, *HSPA1L* and *TAF15*) between groups including family cohesion, family conflict and Global Assessment of Functioning as covariates. Correlations between gene expression and methylation levels were assessed using Pearson's correlation test. Significance was set at *P*<0.05.

## Results

### Sample

Demographic data from the subjects are presented in Table 1. While groups did not differ for age, gender, ethnicity, race, years of education or pubertal development (*P*>0.05 for all comparisons), patients with pediatric BD presented significantly higher scores for manic and depressive symptoms, as well as impairments in functioning when compared to controls and unaffected offspring. In addition, five of the patients were on medication (three on atypical antipsychotics and two on antidepressants).

### Transcriptome profiling

**Figure 1:**
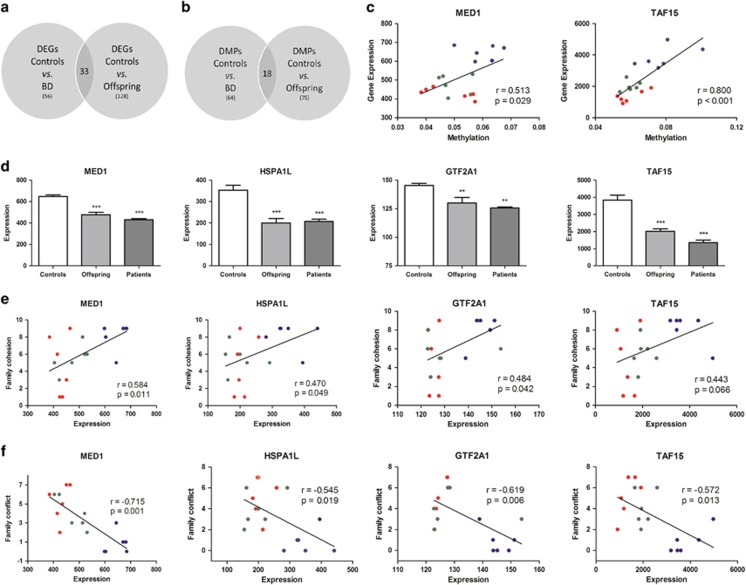
‘Risk genes' identified by the differential expression and methylation analyses. The lists of DEGs (**a**) and DMPs (**b**) between controls versus BD patients and between controls versus offspring were compared to identify concordant genes. Thirty-three genes were concordant in the gene expression analysis, while 18 probes were concordant in the methylation analysis (annotated to 10 genes). (**c**) Correlation between gene expression and methylation at the *MED1* and *TAF15* genes. (**d**) Expression levels of GR signaling pathway risk genes. (**e**) Correlation between expression levels and family cohesion scores for the GR signaling pathway risk genes. (**f**) Correlation between expression levels and family conflict scores for the GR signaling pathway risk genes. **P<0.01; ***P<0.001 when compared to controls. Blue spots represent values for controls, green for offspring and red for patients. BD, bipolar disorder; DEG, differentially expressed gene; DMP, differentially methylated probes; GR, glucocorticoid receptor.

Out of the 34 694 genes interrogated, 128 were found to be differentially expressed between unaffected high-risk offspring and healthy controls (Supplementary Table 1). Fifty-six genes showed a significant difference between healthy controls and BD patients (Supplementary Table 2), whereas 12 genes were found to be differentially expressed between the high-risk offspring and BD patients (Supplementary Table 3). Thirty-three of the genes that were found to be differentially expressed between controls and unaffected high-risk offspring are also in the list of genes that were different between controls and BD patients (Table 2), suggesting them as ‘risk genes' (Figure 1a). Array data were validated by confirming the differences in four selected genes by real-time quantitative PCR (Supplementary Figure S1).

### Methylome profiling

DNA methylation analysis showed that controls and unaffected high-risk offspring differed for 75 probes (Supplementary Table 4), while healthy controls and BD patients showed a difference in 64 probes (Supplementary Table 5). Eighteen probes were concordant between the lists of DMPs in offspring versus controls and BD patients versus controls (Figure 1b). These probes are located within the following 10 annotated genes (Table 2): *PQLC2L*, *PCNX*, *MAGI2*, *HOOK2*, *SLC45A4*, *GLUL*, *PGCP*, *LCE2D*, *NLK* and *ZNF195*, suggesting these *loci* as markers of vulnerability (risk genes). Finally, unaffected offspring and BD patients differed in 47 probes (Supplementary Table 6).

### Identification of risk genes and pathway analysis

**Figure 2:**
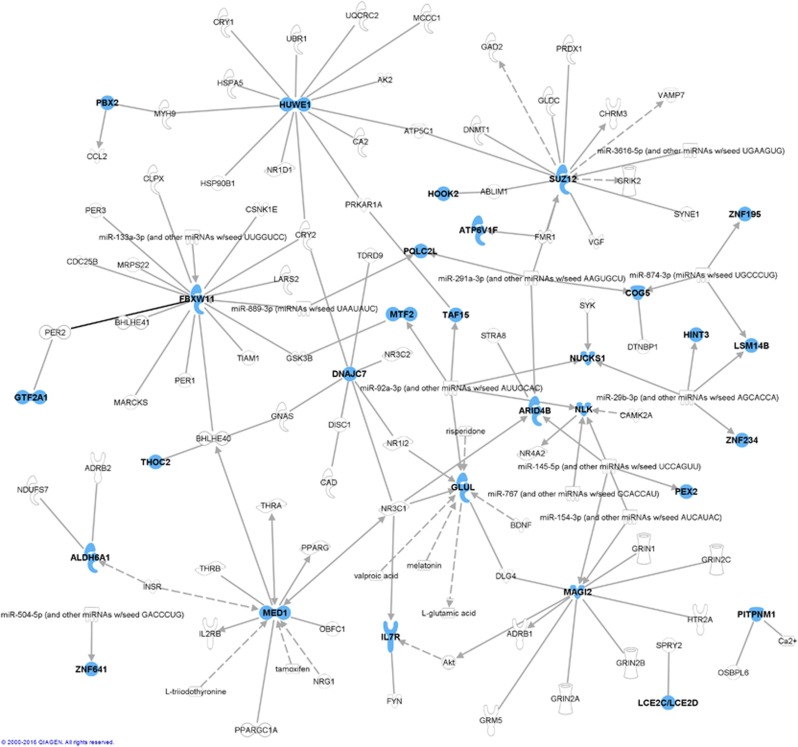
Connections between ‘risk genes' identified in our analysis and molecules previously reported to be associated with bipolar disorder. Molecules painted in blue are ‘risk genes' identified in our analysis, whereas those in grey are known to have been associated with bipolar disorder (available at IPA Knowledge Base). Relationships are based on expression, protein–protein binding, protein–DNA binding, microRNA targeting, activation or transcription. Dashed lines represent indirect relationships (where the two molecules do not need to physically interact). IPA, Ingenuity Pathway Analysis.

We performed pathway analysis on the 43 ‘risk genes' using the IPA software to identify gene networks of potential relevance for BD (Table 3 and Supplementary Table 7). Two genes were not mapped in IPA and therefore were not included in the analysis (*LOC100131360* and *LOC728649*). The top significant canonical pathway enriched in our analysis was the GR signaling pathway (*P*=0.00194), followed by the glutamine biosynthesis I (*P*=0.00198) and the estrogen receptor signaling pathways (*P*=0.00207). Using the IPA Knowledge Base, most of the risk genes were shown to connect directly or indirectly with molecules and networks previously reported to be associated with BD (Figure 2). These gene networks include genes involved in circadian rhythm, immune system and synaptic scaffolding. Evidence of previous reports of association of the risk genes identified in our analyses with BD was further determined by database mining from linkage, genome-wide association study and genome-wide expression study studies (Table 2 and Supplementary Material).

### Differences between patients and high-risk youth

While our study was designed to specifically identify risk markers that would discriminate between controls and patients/high-risk youth (risk genes), we also performed an exploratory analysis with the genes that differentiated patients from high-risk offspring (Supplementary Tables 3 and 6). Specifically, we performed pathway analysis with the 42 genes obtained after combining the lists of genes from the gene expression and DNA methylation analyses. Five of the genes were not mapped on the IPA software (*FLJ40113*, *LOC100130138*, *LOC23117*, *LOC648226* and *LOC729978*), and therefore the analysis was run with the remaining 37 genes. Interestingly, top-ranked canonical pathways include Fcγ receptor-mediated phagocytosis in macrophages and monocytes, PTEN signaling, Cdc42 signaling, Tec kinase signaling and interleukin-15 production, which have all been previously directly or indirectly associated with BD pathophysiology or treatment.^23, 24, 25^

### Correlation between expression and DNA methylation

On the basis of the suggested effects of DNA methylation on the modulation of gene expression, we compared the lists of differentially expressed genes and DMPs for each comparison performed. The only concordance identified was for the gene *LSM5*, which was simultaneously found to be differentially expressed and methylated between controls and BD patients. To further identify which genes were being directly modulated by DNA methylation, we performed an unbiased integrated analysis of gene expression and DNA methylation by correlating all of the genes from both lists, and then filtered the list to identify those correlations with an R Pearson>Abs 0.8. By doing so, we were able to identify genes showing a strong correlation between methylation and expression that had not survived the stringent parameters used for the previous individual analyses (especially considering the fold-change cutoff used for the differential methylation analysis). This analysis identified 135 genes (Supplementary Table 8), among which are the risk genes *FASTK* (*r*=0.85387), *HSPA1L* (*r*=0.86722), *LSM14B* (*r*=−0.83365), *MTF2* (*r*=0.85641), *NUCKS1* (*r*=0.89758), *PBX2* (*r*=−0.92892) and *TAF15* (*r*=0.8004). Of note, the correlations for each of the genes discriminate controls from BD patients and high-risk offspring (Figure 1c shows *TAF15* correlation).

### Modulation of the expression of risk genes in lymphoblastoid cells

**Figure 3:**
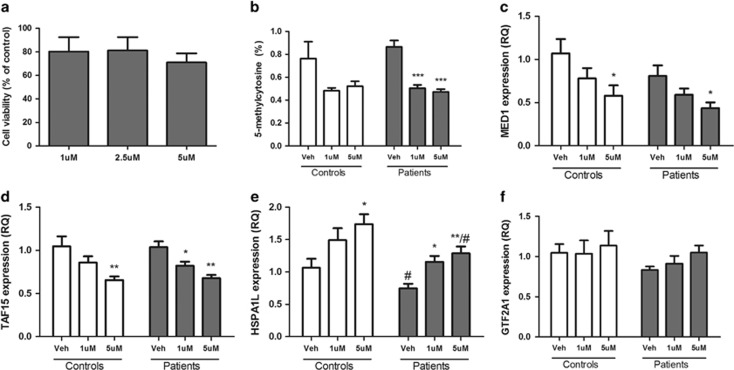
Effects of 5-aza-2'-deoxycytidine treatment in lymphoblastoid cells from adult patients with bipolar disorder and controls. Cells were treated for 96 h (1 or 5 μM). (**a**) Cell viability assessed by MTT assay; (**b**) 5-methylcytosine (%) level; (**c–f**) mRNA levels for *MED1, TAF15, HSPA1L* and *GTF2A1*. *P<0.05, **P<0.01, and ***P<0.001 when compared to vehicle treatment in the same group. ^#^*P*<0.05 when compared to the same treatment in the control group. MTT, (3-(4,5-dimethylthiazol-2-yl)-2,5-diphenyltetrazolium bromide) tetrazolium.

On the basis of the results of the pathway analysis, we sought to functionally investigate the modulation of the four genes assigned to the top-enriched canonical pathway (GR signaling): *MED1*, *GTF2A1*, *HSPA1L* and *TAF15*. Expression of these four genes was reduced in PBMCs from patients with BD and unaffected offspring compared to controls (Figure 1d). To assess the potential role of DNA methylation in modulating the expression of these genes, we measured RNA levels after treating lymphoblastoid cells from adult patients and controls with 5AzadC, a DNA methyltransferase inhibitor. As expected, treatment with 5AzadC significantly reduced global DNA methylation (Figure 3b), whereas no alteration in cell viability was detected (Figure 3a and Supplementary Figure 2). Treatment with 5AzadC significantly reduced the expression levels of *MED1* and *TAF15* and increased the expression of *HSPA1L* in both patients and controls, whereas no statistical differences were found in the expression of *GTF2A1* (Figure 3). Importantly, *HSPA1L* levels were significantly decreased in patients compared to controls irrespective of 5AzadC treatment.

### GR responsiveness in lymphoblastoid cells

**Figure 4:**
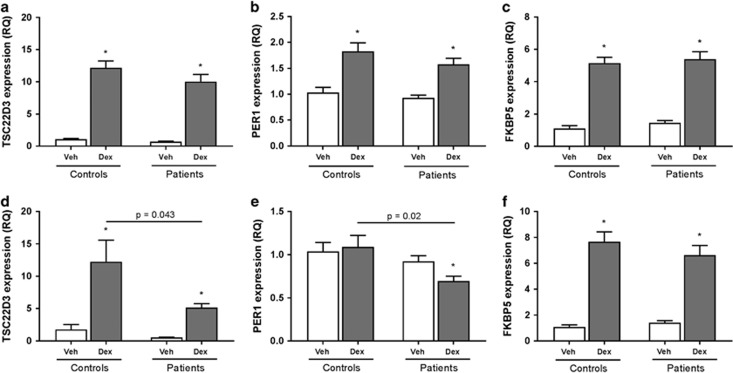
Glucocorticoid receptor-responsive gene expression after stimulation with dexamethasone. Lymphoblastoid cells from adult patients with bipolar disorder and controls were treated with 10^−7^ M for 4 h (**a**–**c**) or 48 h (**d**–**f**) and the expression of glucocorticoid receptor-responsive genes was measured (*TSC22D3*, *PER1* and *FKBP5*). The levels of these genes were also measured by real-time quantitative PCR in PBMCs from children and adolescents that are healthy controls, unaffected offspring of parents with bipolar disorder and patients with pediatric bipolar disorder. *P<0.05 compared to vehicle in the same group. DEX, dexamethasone; PBMC, peripheral blood mononuclear cell; PCR, polymerase chain reaction.

Taking all regulators into account, we sought to investigate parameters related to the GR activity and signaling in the lymphoblastoid cells from adult BD patients and controls. In order to do that, we treated cells *in vitro* with dexamethasone (a GR agonist) for 4 or 48 h and checked for the expression of known GR-responsive genes. Of note, treatment with dexamethasone did not significantly reduce cell viability (Supplementary Figure 3). The use of dexamethasone-induced expression of GR-responsive genes has been shown to successfully predict GR activity in the past.^26, 27^ Specifically, we measured the expression of *FKB5*,^26, 27, 28^ *TSC22D3* (refs 29, 30) and *PER1*,^31^ all of which were responsive to dexamethasone after 4 h of treatment, as expected (Figure 4). No difference between groups was found for the 4-h time point. However, cells from patients showed a significant reduction in the dexamethasone-induced expression of *TSC22D3* (*P*=0.043) and *PER1* (*P*=0.02) after 48 h of treatment compared to controls, while no difference between groups was found for *FKB5* (Figure 4). No difference between patients and controls was seen at baseline for any of the conditions tested. Altogether, these results suggest a subtle yet detectable GR inhibition in adult BD patients compared to controls, which is evident after stimulation with dexamethasone for 48 h.

### Correlation with clinical parameters

Finally, in order to identify the potential impact of the risk genes in clinical measures, we correlated the expression levels of *MED1*, *GTF2A1*, *HSPA1L* and *TAF15* with variables related to family environment and functioning. Family cohesion scores were positively correlated with the expression levels of *HSPA1L* (*P*=0.049), *GTF2A1* (*P*=0.042) and *MED1* (*P*=0.011), but not significantly with *TAF15* (*P*=0.066; Figure 1e). In contrast, family conflict scores were negatively correlated with the expression of the four genes (*HSPA1L*, *P*=0.019; *GTF2A1,P*=0.006; *MED1,* *P*=0.001; *TAF15*, *P*=0.013; Figure 1f). Of note, the correlations for each gene clearly discriminate controls from BD patients and high-risk offspring. In addition to cohesion and conflict, we also found sparse significant correlations between gene expression and the other domains of the Family Environment Scale family, including family independence (MED1, *r*=0.465, *P*=0.046), active-recreational orientation (MED1, *r*=0.515, *P*=0.029; TAF15, *r*=0.501, *P*=0.034) and organization (GTF2A1, *r*=0.528, *P*=0.024). Functioning measures assessed by the Global Assessment of Functioning scale also showed correlations with the expression of *HSPA1L* (*r*=−0.878, *P*=0.021), *MED1* (*r*=0.924, *P*=0.008) and *TAF15* (*r*=0.874, *P*=0.023). Of note, differences between groups in the expression of the GR-related risk genes remained significant even after controlling for functioning, family cohesion and family conflict scores (*MED1*, *F*(2)=13.897, *P*=0.001; *TAF15*, *F*(2)=38.696, *P*<0.001; *HSPA1L*, *F*(2)=16.773, *P*<0.001), except for *GTF2A1* (*F*(2)=3.212, *P*=0.076).

## Discussion

To the best of our knowledge, this is the first study to integrate peripheral genome-wide expression and methylation to identify markers of risk in a population at high risk for BD. Our results show that youth at high risk for BD present common alterations with pediatric BD patients in peripheral gene expression and DNA methylation that differentiate them from healthy controls. A combined analysis of the alterations in expression and methylation led to the identification of 43 ‘risk genes' that are especially enriched for the GR signaling canonical pathway. Our results also show that the expression of the genes assigned to this pathway can be modulated by DNA methylation, which poses the theoretical possibility of manipulating their expression as a means to counteract the familial risk presented by those subjects. Although preliminary, our results suggest the utility of peripheral measures in the identification of biomarkers of risk in high-risk populations and further emphasize the potential role of stress in the risk of BD in youth.

As initially hypothesized, we found that offspring of bipolar parents are much more similar in terms of peripheral expression and methylation events to patients than controls, even when no psychiatric symptoms are yet manifested. Our database and literature mining showed that differential gene expression in peripheral blood cells from BD patients had been previously reported for some of the risk genes identified, including *ZNF641* and *ZNF234*, members of the zinc-finger family of genes, of which *ZNF804A* has been associated with BD and psychosis in genome-wide association study.^32, 33^ Although most of the risk genes had not been previously shown to directly confer risk for BD, many of them are within pathways previously implicated in BD (Figure 2). Our identification of novel genes may be because of the fact that we combined findings from gene expression and DNA methylation. Such a multi-omics approach is crucial not only because of the well-described interplay between DNA methylation and gene expression, but especially when considering the non-canonical roles of DNA methylation (not necessarily altering the expression of the gene at which it is located).^7^

To our surprise, high-risk youth and controls showed a higher number of differentially expressed genes than controls and BD patients. Accordingly, one could hypothesize that most of the gene expression alterations found in the unaffected offspring may account for a compensatory mechanism for the high risk presented by them, ultimately characterizing a resilience factor (of note, all offspring assessed in this study were unaffected for any affective or non-affective psychiatric condition). It is possible that the shift to a full-blown diagnosis might be accompanied by a suppression of these identified expression alterations and the establishment of new illness-specific ones (Supplementary Table S3). Longitudinal studies will be able to clarify this issue.

Interestingly, pathway analysis performed with the risk genes suggested that GR signaling is the top-ranked pathway associated with BD risk in the periphery. This result is in accordance with several studies that suggest that the hypothalamus–pituitary–adrenal (HPA) axis may have a key role in BD and its risk in first-degree relatives.^28^ In fact, HPA dysfunction, along with a dysfunction in circadian rhythm and the immune system, has been proposed as one of the main biological factors underlying the risk for BD in offspring of bipolar parents.^34, 35^ Moreover, high-risk offspring are more likely to have experienced episodic and chronic interpersonal stress,^36^ and they have also been shown to present higher daytime cortisol levels than low-risk offspring.^37, 38, 39^ Prospective studies have also shown that abnormalities in the HPA axis predict the onset of an affective disorder in different samples of high-risk youth, including the offspring of BD parents.^40, 41, 42, 43^ Of note, the alterations found in the expression of the four risk genes assigned to the GR signaling pathway (*HSPA1L*, *TAF15*, *GTF2A1* and *MED1*) might be contributing to this purported HPA axis dysfunction. *HSPA1L* (heat shock 70-kDa protein 1-like) is a member of the heat shock protein 70 family and has been shown to inactivate GR through partial unfolding.^44^ Likewise, *TAF15* (TAF15 RNA polymerase II, TATA box binding protein-associated factor, 68 kDa) and *GTF2A1* (general transcription factor IIA, 1, 19/37 kDa) have also been indirectly related to GR inhibition.^45, 46, 47^ On the contrary, *MED1* (mediator complex subunit 1) has been shown to increase the activation of the glucocorticoid–GR dimer in *nuclei*.^48^ The interplay between these four genes, along with other known stress-responsive genes, might be leading to a GR dysfunction and an increased vulnerability to the long-lasting negative effects of stress.

In this context, we used a cell-based assay to further explore predictors of GR activity and the means by which expression of these GR-related genes can be modulated in BD patients and controls. As hypothesized, taking all regulators into account, our results suggest that GR is slightly hyporesponsive in the cells from adult patients compared to healthy controls, which is in accordance with previous studies.^28^ Moreover, with the exception of *GTF2A1*, our results show that inhibiting DNA methylation can alter the expression of the GR-related risk genes. Specifically, as opposed to what is traditionally thought as the repressive effect of DNA methylation, the expression of *MED1* and *TAF15* was significantly decreased after inhibition of DNA methylation, whereas *HSPA1L* expression was increased after treatment. This indicates that differential methylation patterns might be contributing to the alterations seen in the high-risk youth, and suggests the possibility of targeting this process to prevent illness onset. Of note, *HSPA1L* expression was decreased in the adult bipolar patients compared to controls, as was seen in the pediatric BD and high-risk subjects, further validating a role for this gene in risk for BD.

It is hypothesized that the risk of BD results from the interaction between genetic alterations and environmental factors.^2, 7^ Accordingly, high-risk offspring who experienced high interpersonal chronic stress display a larger cortisol rise following awakening than those reporting low interpersonal chronic stress,^49^ and low levels of structure provided by parents have been predictive of an elevated cortisol response following awakening and during a laboratory psychosocial stressor.^50^ Altogether, these studies suggest an important role of family environment in modulating HPA axis activity in youth at high risk. Accordingly, the four GR-related risk genes identified in our analysis showed significant correlations with family environment scores, most notably family cohesion and conflict, and these correlations clearly discriminate controls from BD patients and high-risk offspring. Therefore, it is possible that the familial risk presented by BD offspring can be counteracted by targeting the environment and thereby possibly the expression of risk genes. In fact, existing family-focused stress-management interventions for children at risk for BD^51^ might have important relevance for future longitudinal studies examining gene expression and methylation in high-risk youth. Noteworthy, we need to consider that other clinical factors might also differentiate the patients and high-risk sample from the controls, including more serious adverse events such as early trauma and maltreatment (which were not available for our particular sample). Future studies should include such measures in the search for risk biomarkers in vulnerable youth.

The results of our study need to be discussed in light of some limitations. First, this is a preliminary cross-sectional analysis with a small sample size, and we cannot rule out the possibility of type I error (false-positives) in our findings. Accordingly, our results must be seen as exploratory and one needs to consider that statistically empowered studies are now required for replication and validation. In this sense, only longitudinal studies with larger sample sizes will be able to determine which of these identified alterations are of significant relevance for illness onset and/or resilience. Moreover, larger studies will also be able to account for the heterogeneity of BD patients when assessing differences in the risk transmitted to their offspring, which is particularly warranted. Of note, in the context of finding relevant markers to be assessed at an individual subject level (personalized medicine), one could argue that these markers should be detectable also in very small sample sizes. This is not to discredit the real limitation of our analysis, but rather to emphasize the potential clinical utility of our results. In this sense, an analysis such as ours is much more likely to identify broad pathways and mechanisms to be followed up in high-risk populations than to really pinpoint definitive specific genes (which would need to be identified at the genome-wide level with much larger sample sizes). Accordingly, our further validation of the GR-related genes with the cell-based assays suggests the relevance and importance of our preliminary results, regardless of the potential limitations inherent to the sample size. Second, given the lack of genotype information, analysis of expression and methylation quantitative trait *loci* was not possible. Both of these analyses would be of importance in future studies in light of the proposed multifactorial model for the risk of complex psychiatric disorders. Third, our search for risk genes was performed in PBMCs, which do not necessarily represent a proxy of brain expression and methylation. Nevertheless, there is evidence showing concordances between both tissues,^52, 53, 54^ and measures in blood have already been correlated with brain volume^10^ and psychiatric symptoms.^12, 55^ Further, peripheral pathways such as inflammatory and metabolic processes have been consistently associated with BD and its risk, and are highly susceptible to exposure to environmental stress. Alterations in the function of genes within these peripheral pathways, including the GR signaling pathway, may lead to an inability to respond appropriately to a given environmental insult, causing behavioral alterations that may lead to the manifestation of symptoms seen in BD. Fourth, as most patients were not drug-free, it is possible that medication use and its duration (which we could not control for) might be inducing changes in some of the gene expression and methylation markers identified. Finally, as our study does not account for cellular heterogeneity in PBMCs, the methylation results may vary.^56^ Moreover, future studies should include next-generation sequencing technologies for the assessment of gene expression, as opposed to array technology, as this would lead to identification of non-coding sequences or splicing- and allele-specific transcripts that might be of special relevance to the risk for BD.

In summary, our preliminary study provides evidence that peripheral gene expression and DNA methylation can discriminate between youth at high risk, patients with BD and healthy controls. Our results require replication and validation in larger cohorts because of our small sample size and the possibility of type I error. With that in mind, this study suggests that such markers might underlie the familial risk of BD shown by high-risk populations. Specifically, alterations related to the GR signaling were observed, which may help explain the HPA axis alterations previously reported in youth at high risk for BD. The strong correlation between genomic and family environment measures suggests that targeting these parameters might be beneficial in preventing illness onset in this population, or may be targets for early intervention.

## Figures and Tables

**Table 1:** Demographic data

*PBMCs*	*Healthy controls (*n=*6)*	*Unaffected offspring (*n=*6)*	*Bipolar disorder patients (*n=*6)*	P*-value*
Age^a^	11.67 (2.3)	10.67 (3.1)	13.33 (2.4)	0.246
Gender (M/F)	3/3	1/5	2/4	0.472
Ethnicity (H/NH)	1/5	2/4	0/6	0.301
Race (W/H/AA)	2/1/3	0/2/4	0/0/6	0.343
Education (years)^a^	5.67 (2.3)	4.83 (4.0)	7 (2.28)	0.446
YMRS^a^	2.33 (3.2)	10.5 (5.8)	27.3 (9.8)	<0.001
Petersen Development Scores^a^	2.6 (0.8)	2.83 (0.4)	2.5 (0.5)	0.652
CDRS^b^	19.25 (4)	20 (4)	42 (16)	0.002
GAF^a^	88.33 (5.4)	89.17 (4.02)	49.83 (2.4)	0.007
				
*FES*				
Cohesion^a^	8.16 (1.6)	5.5 (1.6)	4.66 (3.5)	0.059
Conflict^a^	0.83 (1.1)	4 (1.6)	5.16 (1.9)	0.001
Expressiveness^a^	4.66 (1.5)	5.0 (1.5)	5.16 (1.4)	0.845
Independence^a^	7.16 (1.1)	6.5 (0.8)	5.33 (1.0)	0.022
Achievement orientation^a^	5.83 (0.7)	6.5 (0.5)	5.33 (1.9)	0.301
Intellectual–cultural orientation^a^	6.66 (0.5)	5.5 (1.7)	5.16 (2.1)	0.275
Active-recreational orientation^a^	6.16 (1.7)	3.16 (2.4)	4.16 (2.3)	0.086
Moral-religious emphasis^a^	6.5 (2.5)	7.0 (2.0)	7.16 (1.1)	0.832
Organization^a^	8 (1.09)	5.33 (2.7)	5.83 (2.3)	0.109
Control^a^	6 (0.89)	5.83 (1.1)	5.5 (0.8)	0.673
				
Age at illness onset^a^	N/A	N/A	9.75 (3.6)	
				
*Lymphoblastoid cell lines*	*Healthy controls (*n=*10)*	*Bipolar disorder patients (*n=*10)*	P-*value*	
Age^a^	37.3 (14.15)	43.2 (10.06)	0.297	
Gender (M/F)	5/5	5/5	1.000	
Ethnicity (H/NH)	2/8	3/7	0.606	
Race (W/H/AA)	9/0/1	10/0/0	0.305	

Abbreviations: AA, African American; CDRS, Childhood Depression Rating Scale; FES, Family Environment Scale; GAF, Global Functioning Assessment (last 7 days); H, Hispanic; N/A, not applicable; NH, non-Hispanic; PBMC, peripheral blood mononuclear cell; W, white; YMRS, Young Mania Rating Scale.

a Mean (s.d.).

b Median (interquartile range).

**Table 3:** Pathway analysis of the risk genes for BD

*Canonical pathways*	P*-value*	*Overlap*	*Genes*
Glucocorticoid receptor signaling	1.94E−03	4/270 (0.015)	*MED1, GTF2A1, HSPA1L, TAF15*
Glutamine biosynthesis I	1.98E−03	1/1 (1)	*GLUL*
Estrogen receptor signaling	2.07E−03	3/128 (0.023)	*MED1, GTF2A1, TAF15*
β-alanine degradation I	3.96E−03	1/2 (0.5)	*ALDH6A1*
Assembly of RNA polymerase II complex	4.42E−03	2/50 (0.04)	*GTF2A1, TAF15*
Protein ubiquitination pathway	1.38E−02	3/254 (0.012)	*NADJC7, HSP1L, UBE2E1*
Xenobiotic metabolism signaling	1.41E−02	3-256 (0.012)	*MED1, ALDH6A1, DNAJC7*
Oleate biosynthesis II (animals)	1.97E−03	1/10 (0.1)	*ALDH6A1*
Aryl hydrocarbon receptor signaling	2.94E−02	2/135 (0.015)	*MED1, ALDH6A1*
Valine degradation I	3.51E−02	1/18 (0.056)	*ALDH6A1*
Aldosterone signaling in epithelial cells	3.60E−02	2/151 (0.013)	*DNAJC7, HSPA1L*
DNA methylation and transcriptional repression signaling	3.90E−02	1/20 (0.05)	*ARID4B*
B-cell development	4.47E−02	1/23 (0.043)	*IL7R*

Abbreviation: BD, bipolar disorder.

**Table 2:** Potential risk genes for BD identified in youth at high risk through a combined transcriptome and methylome analysis

*Gene*	*Definition*	*Analysis*	*Locus*	*Linkage*	*GWAS*^a^	*GWES*^b^
*ALDH6A1*	Aldehyde dehydrogenase 6 family, member A1	E	14q24.3	−	−	+
*ARID4B*	AT-rich interactive domain 4B	E	1q42.3	−	−	+
*ATP6V1F*	ATPase, H+ transporting, lysosomal 14 kDa, V1 subunit F	E	7q32.1	−	−	−
*ATP8B2*	ATPase, aminophospholipid transporter, class I, type 8B, member 2	E	1q21.3	−	−	−
*CLASRP*	CLK4−associating serine/arginine-rich protein	E	19q13.32	+	−	+
*CLPTM1*	Cleft lip and palate-associated transmembrane protein 1	E	19q13.32	+	−	+
*COG5*	Component of oligomeric Golgi complex 5	E	7q22.3	−	−	−
*CPQ*	Carboxypeptidase Q	M	8q22.1	−	−	−
*DNAJC7*	DnaJ (Hsp40) homolog, subfamily C, member 7	E	17q21.2	−	−	+
*FASTK*	Fas-activated serine/threonine kinase	E	7q36.1	−	−	−
*FBXW11*	F-box and WD repeat domain containing 11	E	5q35.1	−	−	+
*GLUL*	Glutamate–ammonia ligase	M	1q25.3	−	−	−
*GTF2A1*	General transcription factor IIA, 1, 19/37 kDa	E	14q31.1	−	−	−
*HINT3*	Histidine triad nucleotide-binding protein 3	E	6q22.32	−	−	−
*HOOK2*	Hook microtubule-tethering protein 2	M	19p13.13	−	−	−
*HSPA1L*	Heat shock 70 kDa protein 1-like	E	6p21.33	−	−	−
*HUWE1*	HECT, UBA and WWE domain containing 1, E3 ubiquitin protein ligase	E	Xp11.22	−	−	+
*IL7R*	Interleukin 7 receptor	E	5p13.2	−	−	−
*LCE2D*	Late cornified envelope 2D	M	1q21.3	−	−	−
*LSM14B*	LSM family member 14B	E	20q13.33	−	−	−
*MAGI2*	Membrane-associated guanylate kinase, WW and PDZ domain containing 2	M	7q21.11	+	−	−
*MED1*	Mediator complex subunit 1	E	17q12	−	−	−
*MTF2*	Metal response element-binding transcription factor 2	E	1p22.1	−	−	+
*NLK*	Nemo-like kinase	M	17q11.2	−	−	+
*NUCKS1*	Nuclear casein kinase and cyclin-dependent kinase substrate 1	E	1q32.1	−	−	+
*PBX2*	Pre-B-cell leukemia homeobox 2	E	6p21.32	−	−	−
*PCNX*	Pecanex homolog (Drosophila)	M	14q24.2	−	−	−
*PEX2*	Peroxisomal biogenesis factor 2	E	8q21.13	−	−	−
*PITPNM1*	Phosphatidylinositol transfer protein, membrane-associated 1	E	11q13.2	−	−	−
*PQLC2L (C3orf55)*	PQ loop repeat containing 2-like	M	3q25.32	−	−	−
*PRR14*	Proline-rich 14	E	16p11.2	−	−	+
*SAFB2*	Scaffold attachment factor B2	E	19p13.3	−	−	+
*SLC45A4*	Solute carrier family 45, member 4	M	8q24.3	+	+	+
*SNORA65*	Small nucleolar RNA, H/ACA box 65	E	9q33.3	−	−	−
*SUZ12*	SUZ12 polycomb repressive complex 2 subunit	E	17q11.2	−	−	+
*TAF15*	TAF15 RNA polymerase II, TATA box-binding protein (TBP)-associated factor, 68 kDa	E	17q12	−	−	+
*THOC2*	THO complex 2	E	Xq25	−	−	+
*UBE2E1*	Ubiquitin-conjugating enzyme E2E 1	E	3p24.2	−	−	−
*ZNF195*	Zinc finger 195	M	11p15.4	−	−	−
*ZNF234*	Zinc finger 234	E	19q13.31	+	−	+
*ZNF641*	Zinc finger 641	E	12q13.11	−	−	+

Abbreviations: BD, bipolar disorder; E, expression; GWAS, genome-wide association study; GWES, genome-wide expression study; M, methylation.

a GWAS, results from dbGaP.

b GWES, results from Gene Expression Omnibus (GEO).
