# Supplementary material for: Integrated transcriptome and methylome analysis in youth at high risk for bipolar disorder: a preliminary analysis
Source: Transl Psychiatry. 2017 Mar 14;7(3):e1059–. doi: 10.1038/tp.2017.32 (PMC5416675; doi:10.1038/tp.2017.32)
Supplement: Supplementary Information [file tp201732x1.pdf]

Supplementary Material

Methods

Real-time quantitative PCR

A few genes shown to be differentially expressed between groups were selected for validation by quantitative real-time PCR using TaqMan® predesigned probes and primers (Life Technologies; Carlsbad, CA, USA), as follows: *SNORA65* – Hs03297864_s1; *PITPNM1* – Hs00907527_m1; *PBX2* – Hs01901345; *CREG1* – Hs00355412_m1. Real-time quantitative PCR was also performed to assess the levels of *MED1*, *GTF2A1*, *HSPA1L*, and *TAF15* in the lymphoblastoid cells using the following TaqMan® probes (Life Technologies): *MED1* – Hs01062348_m1; *GTF2A1* – HS01026831_m1; *HSPA1L* – Hs00271466_s1; *TAF15* – Hs00185844_m1; *FKBP5* – Hs01561006_m1; *TSC22D3* – Hs00608272_m1; *PER1* – Hs01092603_m1. Beta-2 microglobulin (*B2M*, Hs00187842) was used as the endogenous control after an initial screening performed with four other genes (*ACTB*, *HPRT1*, *PGK1* and *GAPDH*) and analyzed with NormFinder[1]. Briefly, 600 ng of total RNA were reverse-transcribed to complementary DNA (cDNA) with the High Capacity cDNA Reverse Transcription Kit (Life Technologies) and used as templates in PCR reactions performed with the TaqMan® Gene Expression Master Mix (Life Technologies), according to manufacturer's instructions. Reactions were run in triplicate on a QuantStudio® 7 Flex Real-Time PCR System (Life Technologies) and analyzed by the delta delta Ct method[2].

Methylome profiling

Genome-wide methylation data from the Infinium HumanMethylation 450 BeadChip array (Illumina) were processed using the GenomeStudio V2011.1 software, where quality control checks, normalization (controls), and background adjustment were performed. The control group was used as the reference for the differential methylation analysis, in which Illumina custom was employed as the error model and false discovery rate was computed. Probes with a detection *p*-value > 0.01, as well as those located on the X or

Y chromosomes or those containing single nucleotide polymorphisms, were removed from the analysis, resulting in 385 248 probes.

Database and literature mining

Database and literature mining was performed to check for evidence of associations between the risk genes identified in our analysis and previous studies in BD patients. Specifically: 1) we checked whether the risk genes are located in *loci* previously linked to BD in linkage studies; 2) we looked for previously reported associations of the risk genes with BD in genome-wide association studies (GWAS) by mining the database for Genotypes and Phenotypes (dbGaP; ncbi.nlm.nih.gov/gap/)[3]; and 3) we looked for differences in the expression of those genes in genome-wide expression studies (GWES) comparing BD patients and controls using the Gene Expression Omnibus (GEO) database (ncbi.nlm.nih.gov/geo/), followed by analysis on the GEO2R (ncbi.nlm.nih.gov/geo/geo2r/).

Results

Database and literature mining

Linkage studies

The region encompassing 'risk gene' *SLC45A4*, 8q24, is among the susceptibility *loci* reported for BD with a LOD score of 3.62 in a cohort of 75 BD families[4]. This association was later replicated in a sample of 3 757 individuals of Latino ancestry[5]. Likewise, the region 19q13, which includes the risk genes *CLPTM1* (19q13.32), *CLASRP* (19q13.32), and *ZNF234* (19q13.31) identified in our analysis, has also been linked to BD susceptibility[6, 7] and is proposed to harbor several putative candidate risk genes for BD[6]. Finally, the region 7q21, which includes the risk gene *MAGI2*, was associated with bipolar spectrum disorder in a linkage analysis[8] and also showed significant evidence for linkage with BD in a meta-analysis[9].

GWAS

The only risk gene previously associated with BD in a GWAS study is *SLC45A4*[10], which was identified in the analysis of 7 481 BD patients and 9 250 healthy controls. Specifically, the association with BD was found at an intergenic single nucleotide polymorphism (SNP) (rs782729) located between the *SLC45A4* and *GPR2* genes (location – 8: 142 300 315; $p = 9.00 \times 10^{-6}$).

GWES

Microarray expression profiles were extracted from the publicly available GEO NCBI database, and the search criteria included the Medical Subject Heading (MeSH) term for "bipolar disorder" and "*Homo sapiens*". After analysis on the GEO2R tool, we compared our list of risk genes with the differentially expressed genes in each of the datasets found. The selected datasets were GSE46449, which compared expression in leukocytes from first-episode (n=3) and currently medicated BD patients (n = 26) with matched healthy controls (n=25)[11], and GSE39653, which compared expression in PBMCs from 24 adult healthy controls and 8 adult patients with BD[12]. Among our list of risk genes, differences in the

expression of *NUCKS1*, *ZNF641*, *PRR14*, *SAFB2*, *CLPTM1*, *FBXW11*, *ALDH6A1*, *CLASRP*, *TAF15*, *ARID4B*, *SUZ12*, *SLC45A4*, *DNAJC7*, *NLK*, *HUWE1*, *MTF2*, *ZNF234*, and *THOC2* have been reported in peripheral blood cells between BD patients and controls[11, 12].

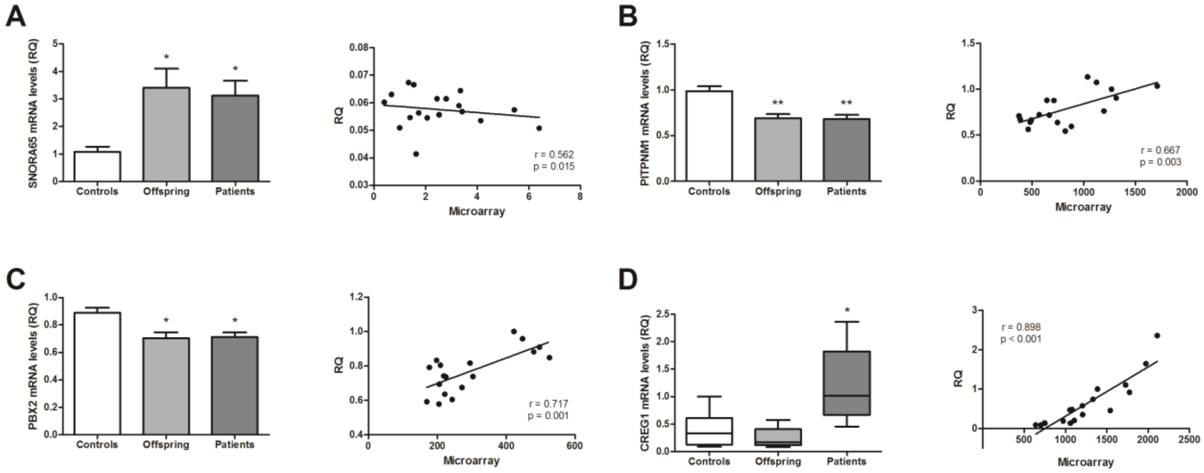

Figure S1. Validation of gene expression microarray results with real-time quantitative PCR. Results from selected risk genes were validated by real-time quantitative PCR using TaqMan® probes and primers: A) *SNORA65*; B) *PIPTNM1*; C) *PBX2*; D) *CREG1*. Expression levels of the target genes were normalized to the expression of beta-2 microglobulin levels, and relative quantification (RQ) levels were determined by the delta delta Ct method[2]. *$p<0.05$; **$p<0.01$ (one-way ANOVA followed by Tukey post-hoc test for results from A-C, and Kruskal-Wallis followed by Mann-Whitney tests for results from D).

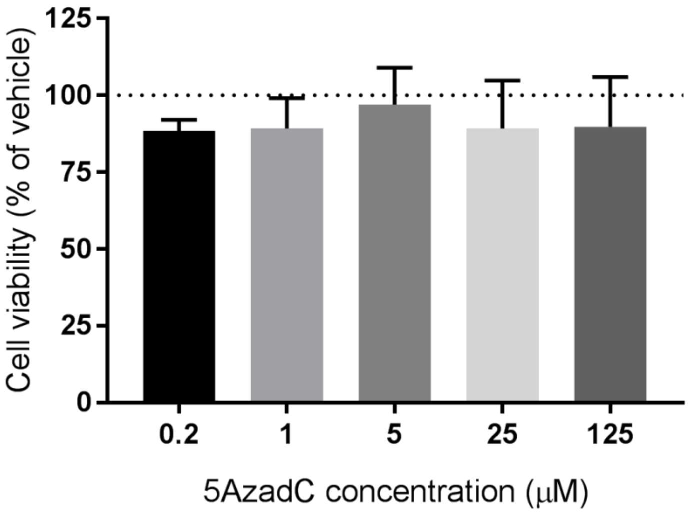

Figure S2. Cell viability after treatment of lymphoblastoid cells from controls (n = 6) with different doses of 5-Aza-2′-deoxycytidine (5-AzadC) for 96 hours. One-way ANOVA $F(5,30) = 1.154$, $p = 0.354$.

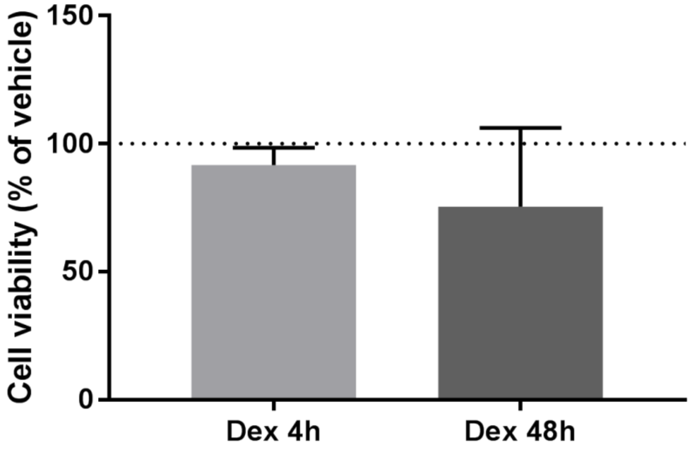

Figure S3. Cell viability after treatment of lymphoblastoid cells from controls (n = 6) with 10^{-7} M dexamethasone for 4 or 48 hours. None of these treatment conditions significantly reduced viability as assessed by MTT reduction. Comparisons between vehicle and dexamethasone were made with Mann-Whitney tests considering Bonferroni-corrected *p*-value < .025 as significant: 4 h – $p = 0.394$; 48 h – $p = 0.066$).

Table S1. Differentially expressed genes between controls and unaffected offspring

Rank	Gene symbol	Probe ID	Mean Controls	Mean Offspring	-log10(*p*-value)
1	*NUCKS1*	4880445	4877.38	2923.15	7.625
2	*PPARBP*	1070195	647.10	429.08	7.165
3	*XPR1*	160441	50.58	76.23	7.007
4	*TAF15*	10176	3835.98	1351.73	6.939
5	*LSM14B*	2360754	22.15	-1.02	6.905
6	*ATP8B2*	6960168	977.42	427.35	6.764
7	*ATP6V1F*	3870630	2322.43	3241.37	6.602
8	*LOC644774*	6580544	852.35	1548.52	6.508
9	*HINT3*	2140279	661.07	2025.78	6.487
10	*IL11RA*	2760148	636.62	288.23	6.450
11	*LOC728649*	4610315	27.70	165.85	6.429
12	*GATAD2A*	1570373	2191.88	1542.20	6.356
13	*NIPSNAP3A*	2490100	178.78	346.08	6.334
14	*THOC2*	2490397	966.20	565.00	6.274
15	*MTF2*	2760255	790.62	560.70	6.201
16	*SFRS16*	2510722	195.30	71.40	6.068
17	*PGK1*	6110400	3213.13	4315.75	6.023
18	*ARID4B*	2470603	586.08	272.12	6.007
19	*CMTM6*	1470520	1911.82	2946.80	5.976
20	*LOC100131360*	1940025	164.93	81.88	5.895
21	*EPC1*	6380008	266.33	105.97	5.885
22	*SAFB2*	2850091	1492.55	1058.78	5.791
23	*PBX2*	5820681	446.10	224.48	5.694
24	*FASTK*	770687	912.35	602.32	5.673
25	*HGS*	5130253	1545.93	1132.97	5.666
26	*TECPR1*	5810196	1139.43	591.27	5.620
27	*HIGD1A*	7100164	725.12	936.85	5.520
28	*DHX37*	4260142	688.78	489.52	5.403
29	*RPS6KA3*	2260133	381.35	192.22	5.320
30	*ZNF364*	7380382	1070.67	823.08	5.296
31	*UBE2E1*	4050070	93.70	50.07	5.289
32	*ZNF641*	6650747	104.62	54.05	5.258
33	*C10ORF26*	150678	142.58	207.30	5.248
34	*KIAA1530*	5910026	333.87	183.22	5.218
35	*SCAP*	3140382	1006.92	774.30	5.194
36	*FBXW11*	3460711	820.33	498.53	5.192
37	*HUWE1*	6980332	144.53	97.85	5.181
38	*ARID4B*	6590722	383.28	219.60	5.166
39	*TOP3A*	3890553	311.82	200.15	5.165
40	*FBXW11*	510253	113.82	61.40	5.148

41	*LENG8*	2000142	82.68	39.95	5.103
42	*SPATA6*	3450193	20.63	53.63	5.052
43	*DDX17*	7000259	399.13	188.72	5.040
44	*SUZ12*	4920619	3164.18	2139.22	5.036
45	*SAFB2*	7050670	1591.58	1101.40	5.001
46	*IL7R*	3830349	3297.30	1441.23	4.994
47	*DNAJC7*	3290037	552.73	351.83	4.969
48	*HIPK2*	10332	553.87	324.13	4.946
49	*FANCD2*	2690240	247.00	124.27	4.930
50	*GMPR2*	2490600	825.77	1051.03	4.889
51	*C1ORF43*	4890113	186.48	312.55	4.886
52	*SERPINB8*	3400672	139.58	265.42	4.879
53	*ZNF275*	3360008	392.43	258.57	4.875
54	*NFATC2IP*	6520201	1067.35	733.93	4.869
55	*CHMP2A*	3290181	150.65	228.52	4.869
56	*ANO9*	5720373	27.58	4.05	4.848
57	*NEURL4*	1990292	78.98	36.20	4.838
58	*BRIP1*	5960730	8.03	31.67	4.829
59	*WDR73*	4210332	110.07	65.82	4.829
60	*BRE*	1400450	372.30	451.30	4.794
61	*TK2*	5690053	51.30	96.08	4.789
62	*PIAS1*	1820019	567.03	420.55	4.780
63	*PCNT*	1510014	710.85	463.02	4.774
64	*PUS1*	60092	1386.98	1058.78	4.769
65	*ALDH6A1*	7100274	116.72	66.72	4.762
66	*COG5*	1430577	203.15	98.00	4.761
67	*FAM32A*	3780440	842.07	1060.72	4.753
68	*LOC100129086*	4760397	331.75	457.12	4.749
69	*ATP6V0E1*	2320110	2541.35	3415.73	4.744
70	*ZNF767*	5090544	75.00	45.78	4.734
71	*CRADD*	1990291	199.03	259.50	4.714
72	*TCERG1*	4490600	41.03	6.92	4.709
73	*PXMP3*	1090288	288.30	428.72	4.704
74	*HNRPDL*	6860678	531.47	332.23	4.704
75	*MARCH3*	780544	441.23	300.97	4.678
76	*LOC646347*	7510209	782.08	1019.82	4.670
77	*SNORA65*	2640019	9.20	72.53	4.669
78	*MRI1*	4860402	97.15	41.12	4.637
79	*LYPLA1*	70300	267.13	399.48	4.623
80	*ACAP1*	7210682	1615.35	1036.08	4.598
81	*PITPNM1*	6960215	1274.90	635.28	4.591
82	*LOC100132707*	6580500	75.28	32.83	4.580
83	*RHEB*	2450139	1227.22	1639.20	4.569

84	*LOC645969*	2750441	173.27	285.58	4.555
85	*GNL3L*	5900746	835.20	525.42	4.542
86	*PRR14*	7570711	1731.32	1435.67	4.525
87	*AZI1*	3390605	211.15	132.97	4.518
88	*TNFSF13*	2140040	51.55	130.52	4.498
89	*ZNF234*	5720072	80.88	44.17	4.498
90	*SEC22C*	7000386	120.37	179.15	4.443
91	*TATDN3*	6020368	139.93	189.23	4.442
92	*MTA2*	2000204	291.53	194.07	4.430
93	*SERP1*	1770541	844.45	1182.87	4.429
94	*PRPS1*	4780521	1013.02	671.53	4.424
95	*ST3GAL1*	1300601	770.65	440.25	4.423
96	*D2HGDH*	2710129	276.40	133.62	4.401
97	*LOC728666*	1110487	390.08	626.28	4.399
98	*EDC4*	6960037	767.75	565.67	4.362
99	*UPF2*	5390603	389.77	233.18	4.349
100	*STMN3*	4780224	2431.45	1385.75	4.346
101	*HSPA1L*	6280471	352.75	207.25	4.341
102	*CD36*	1010592	1330.25	2710.03	4.332
103	*EZR*	2100612	8308.13	6158.52	4.318
104	*CLPTM1*	770168	447.03	293.50	4.291
105	*PLA2G4B*	2350722	857.63	484.65	4.290
106	*RNF217*	6380048	9.48	32.20	4.286
107	*TBC1D10C*	5050603	2504.80	1604.35	4.276
108	*SCNM1*	6350070	256.05	352.42	4.274
109	*C9ORF72*	3990411	110.28	261.98	4.262
110	*GATAD2B*	2370576	365.52	211.90	4.259
111	*ZNF526*	2750040	75.48	41.32	4.251
112	*LSM10*	160255	685.98	927.23	4.244
113	*LOC100128510*	5670431	310.03	149.52	4.244
114	*TTC14*	7000240	313.98	202.08	4.243
115	*ZMYND11*	1190626	2634.08	1754.93	4.239
116	*MTMR14*	2470068	631.07	863.43	4.232
117	*C17ORF41*	6290070	38.35	11.93	4.230
118	*TAF5L*	2320292	131.37	55.27	4.226
119	*SMS*	4490341	979.95	1251.57	4.213
120	*CNIH*	3120767	410.55	598.47	4.198
121	*HS.439031*	2070445	27.83	12.73	4.196
122	*CCS*	2750630	75.03	43.22	4.195
123	*NPIP*	1070070	503.42	303.78	4.194
124	*TAF1C*	6380131	1390.53	811.35	4.179
125	*LOC440731*	4010270	119.50	224.80	4.173
126	*GTF2A1*	5910133	68.88	44.35	4.171

127	*EML3*	3400397	1663.55	1186.53	4.167
128	*NCRNA00120*	7160730	7.82	31.70	4.166

Table S2. Differentially expressed genes between controls and BD patients

Rank	Gene symbol	Probe ID	Mean Controls	Mean BD	-log10(*p*-value)
1	*PAK1IP1*	1990575	199.27	137.17	6.914
2	*UBQLN2*	5560482	1633.40	1082.67	6.560
3	*NUCKS1*	4880445	4877.38	3327.17	6.269
4	*ATP8B2*	6960168	977.42	488.68	6.092
5	*PBX2*	5820681	446.10	215.63	5.908
6	*HINT3*	2140279	661.07	1863.58	5.780
7	*PPARBP*	1070195	647.10	477.17	5.747
8	*ZNF641*	6650747	104.62	49.32	5.733
9	*LOC728649*	4610315	27.70	148.53	5.685
10	*LOC100131360*	1940025	164.93	85.22	5.672
11	*ALDH6A1*	7100274	116.72	57.60	5.626
12	*FBXW11*	3460711	820.33	475.82	5.551
13	*SNORA65*	2640019	9.20	83.18	5.461
14	*COG5*	1430577	203.15	83.68	5.413
15	*SAFB2*	2850091	1492.55	1096.37	5.307
16	*MTP18*	6450184	7.60	34.00	5.302
17	*GTF2A1*	5910133	68.88	38.25	5.259
18	*EEF2*	1580292	15407.50	11164.07	5.230
19	*TAF15*	10176	3835.98	2018.23	5.209
20	*LSM14B*	2360754	22.15	5.20	5.178
21	*CARHSP1*	1230348	1011.63	561.02	5.172
22	*ZNF319*	3180315	527.62	385.93	5.144
23	*MTF2*	2760255	790.62	603.77	5.087
24	*PITPNM1*	6960215	1274.90	569.78	5.077
25	*FASTK*	770687	912.35	635.83	5.072
26	*DNAJC7*	3290037	552.73	349.10	5.037
27	*CLPTM1*	770168	447.03	269.08	5.010
28	*UBE2E1*	4050070	93.70	52.95	4.939
29	*HS.573106*	4390451	10.30	-4.33	4.932
30	*FBXW11*	510253	113.82	64.63	4.826
31	*ZNF234*	5720072	80.88	41.70	4.815
32	*RNY4*	1070079	80.03	347.28	4.794
33	*LOC100129509*	6400343	11.10	-4.87	4.757
34	*ALKBH5*	1400209	6750.58	5578.07	4.735
35	*AP1M1*	2190241	1817.53	1468.32	4.714
36	*MXRA7*	1820181	20.67	0.67	4.713
37	*THOC2*	2490397	966.20	668.52	4.696
38	*PRR14*	7570711	1731.32	1428.45	4.642
39	*SAFB2*	7050670	1591.58	1138.45	4.611
40	*HSPA1L*	6280471	352.75	199.57	4.586

41	*SUZ12*	4920619	3164.18	2236.62	4.541
42	*PXMP3*	1090288	288.30	422.65	4.490
43	*SFRS16*	2510722	195.30	104.02	4.483
44	*C6ORF204*	2000564	204.80	94.43	4.457
45	*HUWE1*	6980332	144.53	104.25	4.449
46	*ALDH6A1*	5820373	226.37	126.45	4.411
47	*ATP6V1F*	3870630	2322.43	2929.05	4.410
48	*IL7R*	3830349	3297.30	1665.37	4.365
49	*ZC3H4*	5820608	1465.00	1010.78	4.339
50	*SNORD101*	3400139	0.55	28.83	4.331
51	*SMURF1*	840598	32.38	68.37	4.303
52	*LSM5*	6650133	324.40	229.03	4.217
53	*NDUFC1*	1110575	1085.17	886.83	4.214
54	*C2ORF27B*	2120446	-5.03	8.32	4.188
55	*MIER3*	6100092	-4.88	10.72	4.167
56	*ARID4B*	2470603	586.08	351.70	3.487

Table S3. Differentially expressed genes between BD patients and unaffected offspring

Rank	Gene symbol	Probe ID	Mean Offspring	Mean BD	-log10(*p*-value)
1	*NIPSNAP3A*	2490100	346.08	214.80	5.028
2	*LOC648226*	4060452	2.18	19.97	4.957
3	*LOC23117*	2850400	2268.88	3798.75	4.614
4	*TNPO2*	2450382	559.57	720.15	4.601
5	*GPR177*	6130136	159.13	50.53	4.520
6	*LOC100130138*	4890653	8.18	27.77	4.497
7	*LOC613037*	2760189	351.17	533.33	4.401
8	*LRP3*	730458	457.68	191.43	4.397
9	*LOC729978*	6980601	2568.68	4299.57	4.389
10	*S100Z*	5870338	187.77	90.42	4.290
11	*FLJ40113*	2320603	-9.07	6.02	4.286
12	*LRP5L*	540403	141.42	276.77	4.269

Table S4. Differentially methylated probes between controls and unaffected offspring

Rank	Index	Target D	Controls AVG Beta	Offspring AVG Beta	Delta Beta	Illumina Gene Annotation
1	10788	cg05593887	0.17765	0.82772	0.65007	*MAGI2*
2	439894	cg25099095	0.71343	0.18537	-0.52806	
3	232939	cg12515659	0.20226	0.6958	0.49354	*FAM134B*
4	244024	cg13232075	0.26133	0.73814	0.4768	
5	164958	cg08522473	0.46799	0.92582	0.45783	*TAGLN3*
6	485539	rs2208123	0.63222	0.18306	-0.44916	
7	420903	cg23943944	0.73918	0.2918	-0.44738	
8	341341	cg18816122	0.10999	0.54603	0.43604	*PLEKHG4B*
9	418678	cg23804921	0.66943	0.23467	-0.43475	
10	125395	cg06417478	0.49136	0.06335	-0.42801	*HOOK2*
11	825	cg00033213	0.76162	0.34312	-0.4185	*TOP1MT*
12	140429	cg07179329	0.81228	0.40225	-0.41004	*CDH13*
13	485542	rs2468330	0.40563	0.80961	0.40398	
14	357637	cg19848924	0.8485	0.44559	-0.40292	
15	214900	cg11418607	0.20225	0.59598	0.39373	
16	485572	rs9292570	0.47504	0.8672	0.39216	
17	1113	cg00045070	0.2168	0.59676	0.37996	*PCSK9*
18	275290	cg14795227	0.66052	0.28297	-0.37755	
19	145092	cg07437919	0.52561	0.14916	-0.37645	*SLC45A4*
20	236587	cg12743416	0.43029	0.05617	-0.37412	*TRIM24*
21	178385	cg09289202	0.32759	0.70086	0.37327	*STK25*
22	151617	cg07791065	0.48947	0.85983	0.37036	
23	297138	cg16147201	0.70586	0.33572	-0.37014	
24	59125	cg02907150	0.42158	0.78546	0.36387	*PCNX*
25	27242	cg01303420	0.73203	0.37015	-0.36188	*C3orf55*
26	214578	cg11401796	0.84504	0.49041	-0.35463	*C21orf70*
27	162978	cg08412936	0.74738	0.40151	-0.34588	*PSORS1C1*
28	163134	cg08422420	0.12593	0.46952	0.34359	*SDHAP3*
29	190626	cg10000195	0.83652	0.49385	-0.34267	
30	220895	cg11792281	0.54053	0.19843	-0.3421	*NLK*
31	424315	cg24136292	0.19632	0.53542	0.33909	*INSC*
32	154968	cg07973095	0.21551	0.55264	0.33713	*DECR2*
33	461936	cg26472636	0.18363	0.52064	0.33701	*SDHAP3*
34	328253	cg18030105	0.6985	0.36216	-0.33634	*ZNF195*
35	157273	cg08104845	0.70519	0.36939	-0.3358	*GLUL*
36	288040	cg15600437	0.87072	0.53831	-0.3324	*MFAP2*
37	182703	cg09533869	0.12189	0.45421	0.33232	*PGCP*
38	169447	cg08778598	0.15171	0.4837	0.33199	*SDHAP3*
39	404358	cg22926869	0.6972	0.36743	-0.32977	*HHLA2*

40	300021	cg16316162	0.03761	0.36569	0.32808	*NAPRT1*
41	377036	cg21117559	0.66503	0.33811	-0.32692	*STARD13*
42	376323	cg21070081	0.51683	0.84299	0.32615	
43	223895	cg11957130	0.61246	0.28831	-0.32415	*ATXN7L1*
44	246480	cg13379757	0.78811	0.46513	-0.32298	
45	452582	cg25929399	0.19389	0.51681	0.32292	*KRT38*
46	192367	cg10098373	0.77258	0.45028	-0.32229	*CPT1A*
47	485554	rs5926356	0.35218	0.67312	0.32094	
48	479924	cg27521571	0.82631	0.50634	-0.31997	*COMT*
49	218975	cg11680857	0.69479	0.37499	-0.3198	*LCE2D*
50	302831	cg16471877	0.6782	0.35857	-0.31963	*COQ3*
51	151367	cg07777042	0.85598	0.53782	-0.31816	
52	282760	cg15251140	0.64789	0.33263	-0.31526	
53	420129	cg23899408	0.47816	0.16322	-0.31494	*HOOK2*
54	389523	cg21931717	0.08616	0.39962	0.31345	*SDHAP3*
55	52721	cg02587993	0.88235	0.56971	-0.31264	
56	321732	cg17605604	0.60023	0.28846	-0.31177	*C5orf30*
57	87981	cg04383836	0.64893	0.33766	-0.31127	*USP43*
58	28217	cg01356752	0.83381	0.52285	-0.31096	
59	237381	cg12798564	0.7643	0.45567	-0.30864	
60	338890	cg18669823	0.62958	0.32139	-0.30819	
61	430609	cg24534774	0.26134	0.56927	0.30793	
62	485521	rs10936224	0.64588	0.33836	-0.30751	
63	485534	rs1941955	0.69993	0.39409	-0.30584	
64	301482	cg16398051	0.77396	0.46897	-0.30499	*ADAMTS17*
65	437392	cg24937727	0.18563	0.49058	0.30495	*RGL3*
66	1158	cg00047185	0.76042	0.45612	-0.3043	*MIR526B*
67	167786	cg08683088	0.72006	0.41583	-0.30424	*ELOVL2*
68	355076	cg19707653	0.7376	0.4353	-0.3023	*KIAA1671*
69	74862	cg03706056	0.23804	0.54032	0.30228	*SETD4*
70	199608	cg10508111	0.8335	0.53137	-0.30212	*PFKM*
71	28700	cg01381374	0.60786	0.30602	-0.30184	
72	100886	cg05059349	0.24652	0.54792	0.3014	
73	108689	cg05477582	0.79757	0.49626	-0.30131	*CMTM1*
74	383609	cg21544585	0.80007	0.49928	-0.30079	*TMEM155; LOC100192379*
75	263104	cg14179288	0.5044	0.80462	0.30022	*TLE4*

Table S5. Differentially methylated probes between controls and BD patients

Rank	Index	Target ID	Controls AVG Beta	BD AVG Beta	Delta Beta	Illumina Gene Annotation
1	348332	cg19285525	0.20847	0.68924	0.48078	*RBMS1*
2	244024	cg13232075	0.26133	0.74139	0.48005	
3	448561	cg25649515	0.44069	0.91658	0.47589	
4	110788	cg05593887	0.17765	0.63184	0.45419	*MAGI2*
5	383107	cg21514997	0.15867	0.5992	0.44054	*CCDC148*
6	125395	cg06417478	0.49136	0.05731	-0.43405	*HOOK2*
7	182703	cg09533869	0.12189	0.54351	0.42162	*PGCP*
8	485542	rs2468330	0.40563	0.80799	0.40235	
9	485561	rs6546473	0.40975	0.8096	0.39985	
10	485566	rs739259	0.22973	0.62793	0.3982	
11	389458	cg21927991	0.85012	0.457	-0.39312	*ZFAT*
12	261966	cg14123034	0.57669	0.18501	-0.39169	*L3MBTL4*
13	485538	rs213028	0.33747	0.72259	0.38512	
14	116088	cg05890457	0.45391	0.83665	0.38274	*ARHGEF10*
15	246480	cg13379757	0.78811	0.40578	-0.38234	
16	485516	rs10457834	0.72943	0.35151	-0.37792	
17	447746	cg25599641	0.92394	0.55674	-0.3672	*NAGA*
18	311144	cg16954525	0.32197	0.68911	0.36713	*MS4A4A*
19	145092	cg07437919	0.52561	0.15922	-0.36639	*SLC45A4*
20	59125	cg02907150	0.42158	0.78787	0.36629	*PCNX*
21	223881	cg11956442	0.41016	0.77368	0.36352	
22	365865	cg20381372	0.33843	0.70088	0.36245	*LOC100134317*
23	367053	cg20459037	0.68591	0.32398	-0.36193	*WDR16*
24	157273	cg08104845	0.70519	0.34573	-0.35946	*GLUL*
25	161458	cg08320316	0.43367	0.78756	0.35389	*RNF216*
26	119400	cg06064179	0.79699	0.44637	-0.35062	*PLEKHM1P*
27	418678	cg23804921	0.66943	0.31977	-0.34966	
28	472472	cg27076160	0.74071	0.39342	-0.34729	*ZNF365*
29	213402	cg11331837	0.42954	0.7728	0.34326	
30	407120	cg23084506	0.54129	0.20045	-0.34084	*PRAMEF5*
31	220895	cg11792281	0.54053	0.20127	-0.33926	*NLK*
32	170144	cg08820231	0.30405	0.64127	0.33723	*SLC26A10*
33	7278	cg00345083	0.30289	0.63962	0.33674	*AJAP1*
34	394844	cg22304519	0.73788	0.40221	-0.33567	
35	485531	rs1510189	0.3942	0.72938	0.33518	
36	420129	cg23899408	0.47816	0.14788	-0.33028	*HOOK2*
37	294557	cg15991478	0.81759	0.48735	-0.33024	*UBD*
38	357637	cg19848924	0.8485	0.51997	-0.32854	
39	96767	cg04850148	0.37416	0.70098	0.32682	*CCL4L2; CCL4L1*

40	146302	cg07501029	0.39536	0.72178	0.32643	*KIF26B*
41	251419	cg13612055	0.49681	0.8211	0.3243	*RNU5E; RNU5D; ACOT12*
42	140933	cg07205452	0.90026	0.57751	-0.32274	*PRDM10*
43	374956	cg20979384	0.28228	0.60258	0.3203	
44	143037	cg07319199	0.83807	0.51884	-0.31923	*ANTXR1*
45	200687	cg10568066	0.3007	0.61986	0.31917	*RNF39*
46	190626	cg10000195	0.83652	0.51888	-0.31764	
47	207438	cg10975354	0.1159	0.43351	0.31761	*VPS13A; LOC100286938*
48	478174	cg27415324	0.72948	0.41529	-0.31419	
49	36864	cg01800061	0.91661	0.60309	-0.31352	*MIR519B; MIR526B*
50	125277	cg06409538	0.54335	0.23001	-0.31334	
51	328253	cg18030105	0.6985	0.38556	-0.31294	*ZNF195*
52	468599	cg26856631	0.50776	0.19611	-0.31164	*LSM5*
53	485565	rs715359	0.66521	0.35434	-0.31088	
54	189001	cg09900440	0.75651	0.44819	-0.30832	*HLA-DPA1*
55	323047	cg17707870	0.12268	0.43009	0.3074	*LHFP*
56	303705	cg16523115	0.59026	0.89605	0.30578	*IL20RB*
57	116629	cg05918715	0.77954	0.47426	-0.30528	*SHISA2*
58	218975	cg11680857	0.69479	0.38959	-0.3052	*LCE2D*
59	275290	cg14795227	0.66052	0.35658	-0.30394	
60	27242	cg01303420	0.73203	0.42815	-0.30389	*C3orf55*
61	485575	rs951295	0.36173	0.66553	0.30379	
62	302713	cg16464924	0.56818	0.87158	0.30339	*GAA*
63	461459	cg26445985	0.34149	0.64327	0.30177	*DENND2A*
64	166866	cg08629394	0.41257	0.71276	0.30018	

Table S6. Differentially methylated probes between unaffected offspring and bipolar disorder patients

Rank	Index	Target ID	Offspring AVG Beta	BD AVG Beta	Delta Beta	Illumina Gene Annotation
1	261966	cg14123034	0.69639	0.18379	-0.5126	*L3MBTL4*
2	232939	cg12515659	0.68621	0.21166	-0.47455	*FAM134B*
3	485539	rs2208123	0.18016	0.6173	0.43714	
4	1113	cg00045070	0.5902	0.16964	-0.42056	*PCSK9*
5	162978	cg08412936	0.39844	0.81823	0.41979	*PSORS1C1*
6	420903	cg23943944	0.29044	0.70992	0.41947	
7	145228	cg07446795	0.87965	0.46651	-0.41313	*CCDC40*
8	140429	cg07179329	0.40153	0.8042	0.40267	*CDH13*
9	116300	cg05900567	0.59664	0.19687	-0.39976	
10	485538	rs213028	0.3321	0.72101	0.38891	
11	460690	cg26398228	0.34913	0.72761	0.37848	
12	407120	cg23084506	0.56815	0.19767	-0.37048	*PRAMEF5*
13	119400	cg06064179	0.79083	0.42867	-0.36217	*PLEKHM1P*
14	485537	rs2125573	0.73229	0.37576	-0.35653	
15	6860	cg00325917	0.48153	0.8371	0.35558	*SELL*
16	164958	cg08522473	0.92101	0.57185	-0.34915	*TAGLN3*
17	179474	cg09351263	0.54725	0.19839	-0.34886	
18	419138	cg23836570	0.5326	0.87885	0.34625	*FBRSL1*
19	213127	cg11314779	0.8831	0.53801	-0.34509	
20	485518	rs10796216	0.55979	0.21687	-0.34292	
21	396292	cg22402398	0.74372	0.4018	-0.34192	*FGR*
22	350115	cg19405842	0.88288	0.54172	-0.34116	*PRKCZ*
23	223881	cg11956442	0.42945	0.76728	0.33783	
24	463816	cg26576978	0.45006	0.78547	0.33541	
25	116108	cg05891136	0.58946	0.92431	0.33486	*MYOM2*
26	476971	cg27341708	0.24295	0.57466	0.33171	*MAML3*
27	202953	cg10701801	0.06483	0.39634	0.33151	*OSBPL9*
28	189001	cg09900440	0.77725	0.44612	-0.33113	*HLA-DPA1*
29	178385	cg09289202	0.69895	0.37018	-0.32877	*STK25*
30	153179	cg07876831	0.76254	0.43471	-0.32783	*TMCO3*
31	301482	cg16398051	0.46819	0.79593	0.32774	*ADAMTS17*
32	66803	cg03299990	0.32387	0.6491	0.32523	
33	235770	cg12687426	0.59724	0.92012	0.32288	*KCNMB3*
34	74862	cg03706056	0.5352	0.21428	-0.32092	*SETD4*
35	348332	cg19285525	0.36288	0.68247	0.3196	*RBMS1*
36	151367	cg07777042	0.52756	0.84707	0.3195	
37	687	cg00028022	0.5443	0.22536	-0.31894	
38	375699	cg21028319	0.67159	0.35529	-0.3163	*TM2D2*

39	311264	cg16963093	0.69048	0.37654	-0.31394	*ATXN7L1*
40	297138	cg16147201	0.33515	0.64603	0.31089	
41	21693	cg01029331	0.61995	0.9284	0.30845	*SDHAP1*
42	485516	rs10457834	0.65718	0.34991	-0.30728	
43	439894	cg25099095	0.1846	0.49178	0.30718	
44	185098	cg09672255	0.53597	0.84182	0.30585	*ZNF879*
45	485572	rs9292570	0.86523	0.56142	-0.30382	
46	36864	cg01800061	0.89669	0.59348	-0.30321	*MIR519B; MIR526B*
47	395319	cg22337626	0.66367	0.361	-0.30266	*MAST2*

Table S7. Top networks enriched in the list of risk genes for BD

ID	Molecules in Network	Score	Focus Molecules	Top Diseases and Functions
1	2010109I03Rik, ADRB2, **ALDH6A1**, APP, **ARID4B**, **ATP6V1F**, beta-estradiol, C14orf93, CASP3, **CLASRP**, **CLPTM1**, **COG5**, FOS, **GLUL**, GPR61, GPR78, GPR85, GPR88, GPR139, GPR173, **HINT3**, **HOOK2**, IGSF6, **LSM14B**, **MAGI2**, MOV10, NTRK1, **NUCKS1**, **PBX2**, **PCNX**, **PEX2**, PIK3R2, **PRR14**, PTTG2, ZNF343	37	16	Cancer, Organismal Injury and Abnormalities, Reproductive System Disease
2	Androgen-AR, CACHD1, CD3, CDC42, CDC42SE1, CDC42SE2, CYP1A1, DNAJB14, **DNAJC7**, DNAJC8, EML4, **FBXW11**, **GTF2A1**, **HSPA1L**, **HUWE1**, **IL7R**, indonel, INF2, LPIN2, MAP2K4/7, **MED1**, **MTF2**, NFkB (complex), POLR2F, POLR2K, RNA pol2-transcription factor, **SAFB2**, **SNORA65**, SUMO4, **SUZ12**, **TAF15**, **THOC2**, TRIM52, ZKSCAN1, **ZNF234**	32	14	Cellular Development, Hematological System Development and Function, Hematopoiesis
3	Anti-inflammatory Cytokine, **ATP8B2**, ATP9A, CHST3, **CPQ**, **FASTK**, GATM, GCC2, GNA14, IL4, ISG15, ITGBL1, ITPA, KITLG, **LCE2C/LCE2D**, LOXL1, LRRC32, METRNL, MNK1/2, MPHOSPH9, MYB, **NLK**, **PITPNM1**, RNF152, PNF220, SCGB3A1, SPRY1, TFAP2A, TGFB1, Tnfrsf22/Tnfrsr23, **UBE2E1**, VTCN1, Zfp35, **ZNF195**, **ZNF641**	19	9	Hematological System Development and Function, Tissue Morphology, Cellular Development
4	LHX1, **SLC45A4**	3	1	Cell Death and Survival, Cellular Movement, Nervous System Development and Function

Table S8. List of genes showing a very strong correlation between expression and methylation ($r^2 > |0.8|$).

Rank	Gene	Pearson's R
1	*HLA-DRB5*	0.9832
2	*HLA-DRB5*	0.97487
3	*HLA-DRB5*	0.95908
4	*RAB34*	-0.94641
5	*RAB34*	-0.94073
6	*RAB34*	-0.94067
7	*HLA-DRB5*	0.93113
8	*PBX2*	-0.92892
9	*KIFC1*	-0.92295
10	*ZNF384*	0.9093
11	*RAB34*	-0.9045
12	*RIMBP2*	-0.89906
13	*NUCKS1*	0.89758
14	*HLA-DRB5*	0.89693
15	*RAB34*	-0.89405
16	*RAB34*	-0.89279
17	*UBAC2*	-0.87961
18	*TADA3*	0.87778
19	*RAB34*	-0.87489
20	*SUPT6H*	-0.87316
21	*PBX2*	-0.86884
22	*CENPT*	0.86767
23	*HLA-DRB5*	0.86722
24	*PGAM4*	0.86576
25	*HSPA1L*	0.86424
26	*PBX2*	-0.86097
27	*RAB43*	-0.86053
28	*NUCKS1*	0.86032
29	*MAP7*	-0.857
30	*MTF2*	0.85641
31	*HLA-DRB5*	0.85522
32	*FASTK*	0.85387
33	*EIF1AY*	-0.85039

Rank	Gene	Pearson's R
34	*MALT1*	0.84992
35	*SFRS18*	0.84987
36	*ANUBL1*	-0.84985
37	*PTPRN2*	-0.84947
38	*PTPRN2*	-0.84947
39	*PSMB8*	0.84916
40	*ADARB2*	-0.84903
41	*GABRG2*	-0.84892
42	*TFDP1*	-0.84777
43	*PRX*	0.84697
44	*FAM13A*	-0.84664
45	*HSPA1L*	0.84606
46	*QKI*	-0.84454
47	*E2F4*	0.8439
48	*ASF1B*	0.84344
49	*MBNL3*	0.84257
50	*ADPGK*	-0.8411
51	*KDM2B*	0.84085
52	*TAF1C*	0.84057
53	*PTPRN2*	-0.83875
54	*ZNF692*	0.83808
55	*BZW2*	0.83703
56	*BCL2*	-0.83665
57	*SFRS18*	0.83653
58	*SEPT9*	-0.83409
59	*LSM14B*	-0.83365
60	*NUCKS1*	-0.83333
61	*METRNL*	0.83154
62	*USP5*	0.83114
63	*EIF1AY*	-0.83085
64	*CD44*	-0.83077
65	*MAML3*	0.83076
66	*MRAS*	-0.82975

Rank	Gene	Pearson's R
67	*TIAF1*	-0.82758
68	*DDX46*	0.82721
69	*LDHB*	0.82517
70	*HLA-DRB5*	-0.82501
71	*KIAA1737*	-0.82487
72	*MYOM1*	0.82189
73	*ZNF384*	0.82185
74	*SAE1*	0.8196
75	*PTPRN2*	-0.81954
76	*CPSF1*	0.81819
77	*NUCKS1*	0.818
78	*ENC1*	0.81718
79	*SLC22A17*	0.81707
80	*CCR4*	0.81637
81	*PRDM16*	-0.81578
82	*DCUN1D4*	-0.81524
83	*MCM6*	0.81513
84	*ITPKB*	-0.81471
85	*SMAD3*	-0.81456
86	*PTTG1*	-0.81433
87	*CSMD1*	-0.81405
88	*PGAM4*	0.8137
89	*KIF17*	-0.81316
90	*RPS6KA3*	-0.81309
91	*PUM1*	0.81179
92	*TIGD1*	0.81148
93	*ZNF92*	-0.81147
94	*SEPT9*	0.81115
95	*RAB11B*	-0.81079
96	*CD248*	-0.8105
97	*PBX2*	-0.80972
98	*EDN3*	-0.80961
99	*ERMN*	-0.80952
100	*EN1*	0.80884

Rank	Gene	Pearson's R
101	*KIAA1530*	0.80869
102	*PCNA*	-0.80854
103	*HOXC4;HOXC6;HOXC5*	0.80815
104	*C19orf6*	0.80808
105	*CTDP1*	-0.80797
106	*MAP7*	-0.80708
107	*RNF212*	-0.80683
108	*UGP2*	0.80669
109	*BAT2*	0.80626
110	*FTH1*	-0.80615
111	*NSUN5*	-0.80603
112	*ACTN1*	-0.80561
113	*RWDD1*	0.80555
114	*ATP5D*	0.80539
115	*MAP4K4*	0.80475
116	*MGST1*	-0.80432
117	*NUCKS1*	0.8043
118	*ADPRHL1*	-0.80421
119	*ATP11A*	-0.80337
120	*IQCK*	0.80307
121	*SEC14L1*	-0.80244
122	*KIAA1407*	0.80221
123	*FAM98A*	-0.80198
124	*HNRNPUL2;TTC9C*	0.8019
125	*SCRIB*	-0.80156
126	*SLC29A1*	-0.80153
127	*GSTTP2*	-0.80141
128	*HNRNPA0*	-0.80124
129	*FBXL19*	-0.80119
130	*PHF5A;ACO2*	0.801
131	*CENPT*	0.80095
132	*HSPB1*	-0.80068
133	*C14orf119;ACIN1*	0.80065
134	*USF2*	0.80037
135	*TAF15*	0.80004

References

1. Andersen CL, Jensen JL, Orntoft TF. Normalization of real-time quantitative reverse transcription-PCR data: a model-based variance estimation approach to identify genes suited for normalization, applied to bladder and colon cancer data sets. *Cancer Res* 2004; **64**:5245-50.
2. Livak KJ, Schmittgen TD. Analysis of relative gene expression data using real-time quantitative PCR and the 2(-Delta Delta C(T)) Method. *Methods* 2001; **25**:402-8.
3. Tryka KA, Hao L, Sturcke A, Jin Y, Wang ZY, Ziyabari L, et al. NCBI's Database of Genotypes and Phenotypes: dbGaP. *Nucleic Acids Res* 2014; **42**:D975-9.
4. Cichon S, Schumacher J, Muller DJ, Hurter M, Windemuth C, Strauch K, et al. A genome screen for genes predisposing to bipolar affective disorder detects a new susceptibility locus on 8q. *Hum Mol Genet* 2001; **10**:2933-44.
5. Gonzalez S, Camarillo C, Rodriguez M, Ramirez M, Zavala J, Armas R, et al. A genome-wide linkage scan of bipolar disorder in Latino families identifies susceptibility loci at 8q24 and 14q32. *Am J Med Genet B Neuropsychiatr Genet* 2014; **165b**:479-91.
6. Lekman M, Karlsson R, Graae L, Hossjer O, Kockum I. A significant risk locus on 19q13 for bipolar disorder identified using a combined genome-wide linkage and copy number variation analysis. *BioData Min* 2015; **8**:42.
7. Francks C, Tozzi F, Farmer A, Vincent JB, Rujescu D, St Clair D, et al. Population-based linkage analysis of schizophrenia and bipolar case-control cohorts identifies a potential susceptibility locus on 19q13. *Mol Psychiatry* 2010; **15**:319-25.
8. Georgi B, Craig D, Kember RL, Liu W, Lindquist I, Nasser S, et al. Genomic view of bipolar disorder revealed by whole genome sequencing in a genetic isolate. *PLoS Genet* 2014; **10**:e1004229.
9. Badner JA, Gershon ES. Meta-analysis of whole-genome linkage scans of bipolar disorder and schizophrenia. *Mol Psychiatry* 2002; **7**:405-11.

10. Large-scale genome-wide association analysis of bipolar disorder identifies a new susceptibility locus near ODZ4. *Nat Genet* 2011; **43**:977-83.

11. Clelland CL, Read LL, Panek LJ, Nadrich RH, Bancroft C, Clelland JD. Utilization of never-medicated bipolar disorder patients towards development and validation of a peripheral biomarker profile. *PLoS One* 2013; **8**:e69082.

12. Savitz J, Frank MB, Victor T, Bebak M, Marino JH, Bellgowan PS, et al. Inflammation and neurological disease-related genes are differentially expressed in depressed patients with mood disorders and correlate with morphometric and functional imaging abnormalities. *Brain Behav Immun* 2013; **31**:161-71.

Supplementary Material

Methods

Real-time quantitative PCR

A few genes shown to be differentially expressed between groups were selected for validation by quantitative real-time PCR using TaqMan® predesigned probes and primers (Life Technologies; Carlsbad, CA, USA), as follows: *SNORA65* – Hs03297864_s1; *PITPNM1* – Hs00907527_m1; *PBX2* – Hs01901345; *CREG1* – Hs00355412_m1. Real-time quantitative PCR was also performed to assess the levels of *MED1*, *GTF2A1*, *HSPA1L*, and *TAF15* in the lymphoblastoid cells using the following TaqMan® probes (Life Technologies): *MED1* – Hs01062348_m1; *GTF2A1* – HS01026831_m1; *HSPA1L* – Hs00271466_s1; *TAF15* – Hs00185844_m1; *FKBP5* – Hs01561006_m1; *TSC22D3* – Hs00608272_m1; *PER1* – Hs01092603_m1. Beta-2 microglobulin (*B2M*, Hs00187842) was used as the endogenous control after an initial screening performed with four other genes (*ACTB*, *HPRT1*, *PGK1* and *GAPDH*) and analyzed with NormFinder[1]. Briefly, 600 ng of total RNA were reverse-transcribed to complementary DNA (cDNA) with the High Capacity cDNA Reverse Transcription Kit (Life Technologies) and used as templates in PCR reactions performed with the TaqMan® Gene Expression Master Mix (Life Technologies), according to manufacturer's instructions. Reactions were run in triplicate on a QuantStudio® 7 Flex Real-Time PCR System (Life Technologies) and analyzed by the delta delta Ct method[2].

Methylome profiling

Genome-wide methylation data from the Infinium HumanMethylation 450 BeadChip array (Illumina) were processed using the GenomeStudio V2011.1 software, where quality control checks, normalization (controls), and background adjustment were performed. The control group was used as the reference for the differential methylation analysis, in which Illumina custom was employed as the error model and false discovery rate was computed. Probes with a detection *p*-value > 0.01, as well as those located on the X or

Y chromosomes or those containing single nucleotide polymorphisms, were removed from the analysis, resulting in 385 248 probes.

Database and literature mining

Database and literature mining was performed to check for evidence of associations between the risk genes identified in our analysis and previous studies in BD patients. Specifically: 1) we checked whether the risk genes are located in *loci* previously linked to BD in linkage studies; 2) we looked for previously reported associations of the risk genes with BD in genome-wide association studies (GWAS) by mining the database for Genotypes and Phenotypes (dbGaP; ncbi.nlm.nih.gov/gap/)[3]; and 3) we looked for differences in the expression of those genes in genome-wide expression studies (GWES) comparing BD patients and controls using the Gene Expression Omnibus (GEO) database (ncbi.nlm.nih.gov/geo/), followed by analysis on the GEO2R (ncbi.nlm.nih.gov/geo/geo2r/).

Results

Database and literature mining

Linkage studies

The region encompassing 'risk gene' *SLC45A4*, 8q24, is among the susceptibility *loci* reported for BD with a LOD score of 3.62 in a cohort of 75 BD families[4]. This association was later replicated in a sample of 3 757 individuals of Latino ancestry[5]. Likewise, the region 19q13, which includes the risk genes *CLPTM1* (19q13.32), *CLASRP* (19q13.32), and *ZNF234* (19q13.31) identified in our analysis, has also been linked to BD susceptibility[6, 7] and is proposed to harbor several putative candidate risk genes for BD[6]. Finally, the region 7q21, which includes the risk gene *MAGI2*, was associated with bipolar spectrum disorder in a linkage analysis[8] and also showed significant evidence for linkage with BD in a meta-analysis[9].

GWAS

The only risk gene previously associated with BD in a GWAS study is *SLC45A4*[10], which was identified in the analysis of 7 481 BD patients and 9 250 healthy controls. Specifically, the association with BD was found at an intergenic single nucleotide polymorphism (SNP) (rs782729) located between the *SLC45A4* and *GPR2* genes (location – 8: 142 300 315; $p = 9.00 \times 10^{-6}$).

GWES

Microarray expression profiles were extracted from the publicly available GEO NCBI database, and the search criteria included the Medical Subject Heading (MeSH) term for "bipolar disorder" and "*Homo sapiens*". After analysis on the GEO2R tool, we compared our list of risk genes with the differentially expressed genes in each of the datasets found. The selected datasets were GSE46449, which compared expression in leukocytes from first-episode (n=3) and currently medicated BD patients (n = 26) with matched healthy controls (n=25)[11], and GSE39653, which compared expression in PBMCs from 24 adult healthy controls and 8 adult patients with BD[12]. Among our list of risk genes, differences in the

expression of *NUCKS1*, *ZNF641*, *PRR14*, *SAFB2*, *CLPTM1*, *FBXW11*, *ALDH6A1*, *CLASRP*, *TAF15*, *ARID4B*, *SUZ12*, *SLC45A4*, *DNAJC7*, *NLK*, *HUWE1*, *MTF2*, *ZNF234*, and *THOC2* have been reported in peripheral blood cells between BD patients and controls[11, 12].

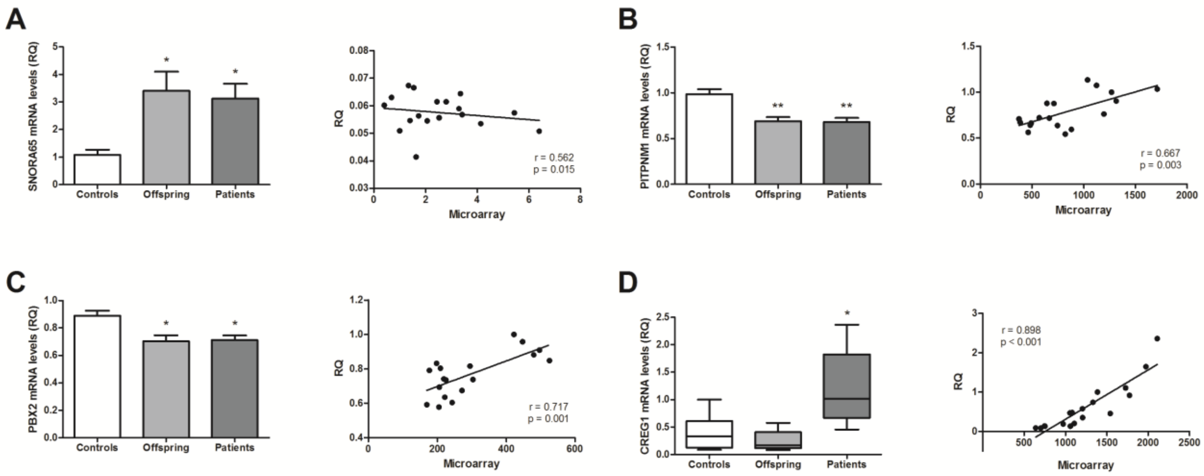

Figure S1. Validation of gene expression microarray results with real-time quantitative PCR. Results from selected risk genes were validated by real-time quantitative PCR using TaqMan® probes and primers: A) *SNORA65*; B) *PIPTNM1*; C) *PBX2*; D) *CREG1*. Expression levels of the target genes were normalized to the expression of beta-2 microglobulin levels, and relative quantification (RQ) levels were determined by the delta delta Ct method[2]. *$p<0.05$; **$p<0.01$ (one-way ANOVA followed by Tukey post-hoc test for results from A-C, and Kruskal-Wallis followed by Mann-Whitney tests for results from D).

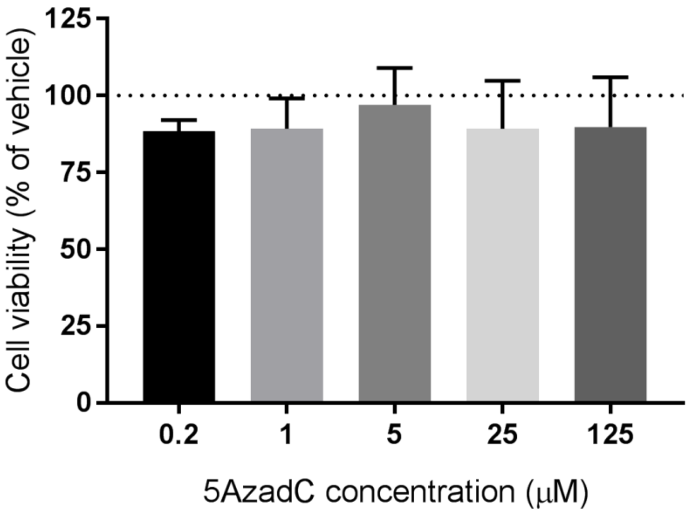

Figure S2. Cell viability after treatment of lymphoblastoid cells from controls (n = 6) with different doses of 5-Aza-2′-deoxycytidine (5-AzadC) for 96 hours. One-way ANOVA $F(5,30) = 1.154$, $p = 0.354$.

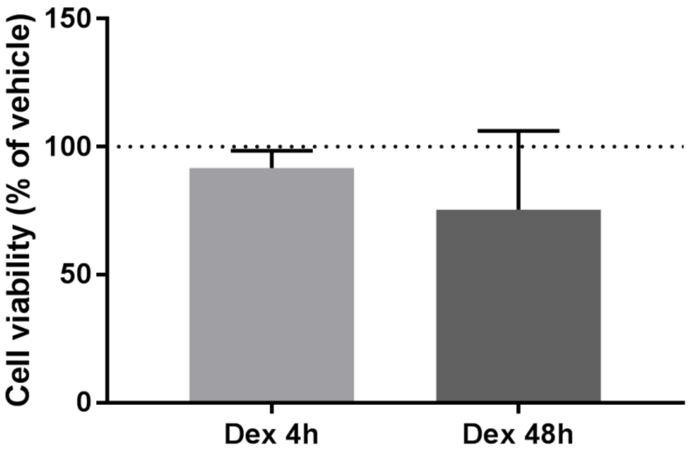

Figure S3. Cell viability after treatment of lymphoblastoid cells from controls (n = 6) with 10^{-7} M dexamethasone for 4 or 48 hours. None of these treatment conditions significantly reduced viability as assessed by MTT reduction. Comparisons between vehicle and dexamethasone were made with Mann-Whitney tests considering Bonferroni-corrected *p*-value < .025 as significant: 4 h – *p* = 0.394; 48 h – *p* = 0.066).

Table S1. Differentially expressed genes between controls and unaffected offspring

Rank	Gene symbol	Probe ID	Mean Controls	Mean Offspring	-log10(*p*-value)
1	*NUCKS1*	4880445	4877.38	2923.15	7.625
2	*PPARBP*	1070195	647.10	429.08	7.165
3	*XPR1*	160441	50.58	76.23	7.007
4	*TAF15*	10176	3835.98	1351.73	6.939
5	*LSM14B*	2360754	22.15	-1.02	6.905
6	*ATP8B2*	6960168	977.42	427.35	6.764
7	*ATP6V1F*	3870630	2322.43	3241.37	6.602
8	*LOC644774*	6580544	852.35	1548.52	6.508
9	*HINT3*	2140279	661.07	2025.78	6.487
10	*IL11RA*	2760148	636.62	288.23	6.450
11	*LOC728649*	4610315	27.70	165.85	6.429
12	*GATAD2A*	1570373	2191.88	1542.20	6.356
13	*NIPSNAP3A*	2490100	178.78	346.08	6.334
14	*THOC2*	2490397	966.20	565.00	6.274
15	*MTF2*	2760255	790.62	560.70	6.201
16	*SFRS16*	2510722	195.30	71.40	6.068
17	*PGK1*	6110400	3213.13	4315.75	6.023
18	*ARID4B*	2470603	586.08	272.12	6.007
19	*CMTM6*	1470520	1911.82	2946.80	5.976
20	*LOC100131360*	1940025	164.93	81.88	5.895
21	*EPC1*	6380008	266.33	105.97	5.885
22	*SAFB2*	2850091	1492.55	1058.78	5.791
23	*PBX2*	5820681	446.10	224.48	5.694
24	*FASTK*	770687	912.35	602.32	5.673
25	*HGS*	5130253	1545.93	1132.97	5.666
26	*TECPR1*	5810196	1139.43	591.27	5.620
27	*HIGD1A*	7100164	725.12	936.85	5.520
28	*DHX37*	4260142	688.78	489.52	5.403
29	*RPS6KA3*	2260133	381.35	192.22	5.320
30	*ZNF364*	7380382	1070.67	823.08	5.296
31	*UBE2E1*	4050070	93.70	50.07	5.289
32	*ZNF641*	6650747	104.62	54.05	5.258
33	*C10ORF26*	150678	142.58	207.30	5.248
34	*KIAA1530*	5910026	333.87	183.22	5.218
35	*SCAP*	3140382	1006.92	774.30	5.194
36	*FBXW11*	3460711	820.33	498.53	5.192
37	*HUWE1*	6980332	144.53	97.85	5.181
38	*ARID4B*	6590722	383.28	219.60	5.166
39	*TOP3A*	3890553	311.82	200.15	5.165
40	*FBXW11*	510253	113.82	61.40	5.148

41	*LENG8*	2000142	82.68	39.95	5.103
42	*SPATA6*	3450193	20.63	53.63	5.052
43	*DDX17*	7000259	399.13	188.72	5.040
44	*SUZ12*	4920619	3164.18	2139.22	5.036
45	*SAFB2*	7050670	1591.58	1101.40	5.001
46	*IL7R*	3830349	3297.30	1441.23	4.994
47	*DNAJC7*	3290037	552.73	351.83	4.969
48	*HIPK2*	10332	553.87	324.13	4.946
49	*FANCD2*	2690240	247.00	124.27	4.930
50	*GMPR2*	2490600	825.77	1051.03	4.889
51	*C1ORF43*	4890113	186.48	312.55	4.886
52	*SERPINB8*	3400672	139.58	265.42	4.879
53	*ZNF275*	3360008	392.43	258.57	4.875
54	*NFATC2IP*	6520201	1067.35	733.93	4.869
55	*CHMP2A*	3290181	150.65	228.52	4.869
56	*ANO9*	5720373	27.58	4.05	4.848
57	*NEURL4*	1990292	78.98	36.20	4.838
58	*BRIP1*	5960730	8.03	31.67	4.829
59	*WDR73*	4210332	110.07	65.82	4.829
60	*BRE*	1400450	372.30	451.30	4.794
61	*TK2*	5690053	51.30	96.08	4.789
62	*PIAS1*	1820019	567.03	420.55	4.780
63	*PCNT*	1510014	710.85	463.02	4.774
64	*PUS1*	60092	1386.98	1058.78	4.769
65	*ALDH6A1*	7100274	116.72	66.72	4.762
66	*COG5*	1430577	203.15	98.00	4.761
67	*FAM32A*	3780440	842.07	1060.72	4.753
68	*LOC100129086*	4760397	331.75	457.12	4.749
69	*ATP6V0E1*	2320110	2541.35	3415.73	4.744
70	*ZNF767*	5090544	75.00	45.78	4.734
71	*CRADD*	1990291	199.03	259.50	4.714
72	*TCERG1*	4490600	41.03	6.92	4.709
73	*PXMP3*	1090288	288.30	428.72	4.704
74	*HNRPDL*	6860678	531.47	332.23	4.704
75	*MARCH3*	780544	441.23	300.97	4.678
76	*LOC646347*	7510209	782.08	1019.82	4.670
77	*SNORA65*	2640019	9.20	72.53	4.669
78	*MRI1*	4860402	97.15	41.12	4.637
79	*LYPLA1*	70300	267.13	399.48	4.623
80	*ACAP1*	7210682	1615.35	1036.08	4.598
81	*PITPNM1*	6960215	1274.90	635.28	4.591
82	*LOC100132707*	6580500	75.28	32.83	4.580
83	*RHEB*	2450139	1227.22	1639.20	4.569

84	*LOC645969*	2750441	173.27	285.58	4.555
85	*GNL3L*	5900746	835.20	525.42	4.542
86	*PRR14*	7570711	1731.32	1435.67	4.525
87	*AZI1*	3390605	211.15	132.97	4.518
88	*TNFSF13*	2140040	51.55	130.52	4.498
89	*ZNF234*	5720072	80.88	44.17	4.498
90	*SEC22C*	7000386	120.37	179.15	4.443
91	*TATDN3*	6020368	139.93	189.23	4.442
92	*MTA2*	2000204	291.53	194.07	4.430
93	*SERP1*	1770541	844.45	1182.87	4.429
94	*PRPS1*	4780521	1013.02	671.53	4.424
95	*ST3GAL1*	1300601	770.65	440.25	4.423
96	*D2HGDH*	2710129	276.40	133.62	4.401
97	*LOC728666*	1110487	390.08	626.28	4.399
98	*EDC4*	6960037	767.75	565.67	4.362
99	*UPF2*	5390603	389.77	233.18	4.349
100	*STMN3*	4780224	2431.45	1385.75	4.346
101	*HSPA1L*	6280471	352.75	207.25	4.341
102	*CD36*	1010592	1330.25	2710.03	4.332
103	*EZR*	2100612	8308.13	6158.52	4.318
104	*CLPTM1*	770168	447.03	293.50	4.291
105	*PLA2G4B*	2350722	857.63	484.65	4.290
106	*RNF217*	6380048	9.48	32.20	4.286
107	*TBC1D10C*	5050603	2504.80	1604.35	4.276
108	*SCNM1*	6350070	256.05	352.42	4.274
109	*C9ORF72*	3990411	110.28	261.98	4.262
110	*GATAD2B*	2370576	365.52	211.90	4.259
111	*ZNF526*	2750040	75.48	41.32	4.251
112	*LSM10*	160255	685.98	927.23	4.244
113	*LOC100128510*	5670431	310.03	149.52	4.244
114	*TTC14*	7000240	313.98	202.08	4.243
115	*ZMYND11*	1190626	2634.08	1754.93	4.239
116	*MTMR14*	2470068	631.07	863.43	4.232
117	*C17ORF41*	6290070	38.35	11.93	4.230
118	*TAF5L*	2320292	131.37	55.27	4.226
119	*SMS*	4490341	979.95	1251.57	4.213
120	*CNIH*	3120767	410.55	598.47	4.198
121	*HS.439031*	2070445	27.83	12.73	4.196
122	*CCS*	2750630	75.03	43.22	4.195
123	*NPIP*	1070070	503.42	303.78	4.194
124	*TAF1C*	6380131	1390.53	811.35	4.179
125	*LOC440731*	4010270	119.50	224.80	4.173
126	*GTF2A1*	5910133	68.88	44.35	4.171

127	*EML3*	3400397	1663.55	1186.53	4.167
128	*NCRNA00120*	7160730	7.82	31.70	4.166

Table S2. Differentially expressed genes between controls and BD patients

Rank	Gene symbol	Probe ID	Mean Controls	Mean BD	-log10(*p*-value)
1	*PAK1IP1*	1990575	199.27	137.17	6.914
2	*UBQLN2*	5560482	1633.40	1082.67	6.560
3	*NUCKS1*	4880445	4877.38	3327.17	6.269
4	*ATP8B2*	6960168	977.42	488.68	6.092
5	*PBX2*	5820681	446.10	215.63	5.908
6	*HINT3*	2140279	661.07	1863.58	5.780
7	*PPARBP*	1070195	647.10	477.17	5.747
8	*ZNF641*	6650747	104.62	49.32	5.733
9	*LOC728649*	4610315	27.70	148.53	5.685
10	*LOC100131360*	1940025	164.93	85.22	5.672
11	*ALDH6A1*	7100274	116.72	57.60	5.626
12	*FBXW11*	3460711	820.33	475.82	5.551
13	*SNORA65*	2640019	9.20	83.18	5.461
14	*COG5*	1430577	203.15	83.68	5.413
15	*SAFB2*	2850091	1492.55	1096.37	5.307
16	*MTP18*	6450184	7.60	34.00	5.302
17	*GTF2A1*	5910133	68.88	38.25	5.259
18	*EEF2*	1580292	15407.50	11164.07	5.230
19	*TAF15*	10176	3835.98	2018.23	5.209
20	*LSM14B*	2360754	22.15	5.20	5.178
21	*CARHSP1*	1230348	1011.63	561.02	5.172
22	*ZNF319*	3180315	527.62	385.93	5.144
23	*MTF2*	2760255	790.62	603.77	5.087
24	*PITPNM1*	6960215	1274.90	569.78	5.077
25	*FASTK*	770687	912.35	635.83	5.072
26	*DNAJC7*	3290037	552.73	349.10	5.037
27	*CLPTM1*	770168	447.03	269.08	5.010
28	*UBE2E1*	4050070	93.70	52.95	4.939
29	*HS.573106*	4390451	10.30	-4.33	4.932
30	*FBXW11*	510253	113.82	64.63	4.826
31	*ZNF234*	5720072	80.88	41.70	4.815
32	*RNY4*	1070079	80.03	347.28	4.794
33	*LOC100129509*	6400343	11.10	-4.87	4.757
34	*ALKBH5*	1400209	6750.58	5578.07	4.735
35	*AP1M1*	2190241	1817.53	1468.32	4.714
36	*MXRA7*	1820181	20.67	0.67	4.713
37	*THOC2*	2490397	966.20	668.52	4.696
38	*PRR14*	7570711	1731.32	1428.45	4.642
39	*SAFB2*	7050670	1591.58	1138.45	4.611
40	*HSPA1L*	6280471	352.75	199.57	4.586

41	*SUZ12*	4920619	3164.18	2236.62	4.541
42	*PXMP3*	1090288	288.30	422.65	4.490
43	*SFRS16*	2510722	195.30	104.02	4.483
44	*C6ORF204*	2000564	204.80	94.43	4.457
45	*HUWE1*	6980332	144.53	104.25	4.449
46	*ALDH6A1*	5820373	226.37	126.45	4.411
47	*ATP6V1F*	3870630	2322.43	2929.05	4.410
48	*IL7R*	3830349	3297.30	1665.37	4.365
49	*ZC3H4*	5820608	1465.00	1010.78	4.339
50	*SNORD101*	3400139	0.55	28.83	4.331
51	*SMURF1*	840598	32.38	68.37	4.303
52	*LSM5*	6650133	324.40	229.03	4.217
53	*NDUFC1*	1110575	1085.17	886.83	4.214
54	*C2ORF27B*	2120446	-5.03	8.32	4.188
55	*MIER3*	6100092	-4.88	10.72	4.167
56	*ARID4B*	2470603	586.08	351.70	3.487

Table S3. Differentially expressed genes between BD patients and unaffected offspring

Rank	Gene symbol	Probe ID	Mean Offspring	Mean BD	-log10(*p*-value)
1	*NIPSNAP3A*	2490100	346.08	214.80	5.028
2	*LOC648226*	4060452	2.18	19.97	4.957
3	*LOC23117*	2850400	2268.88	3798.75	4.614
4	*TNPO2*	2450382	559.57	720.15	4.601
5	*GPR177*	6130136	159.13	50.53	4.520
6	*LOC100130138*	4890653	8.18	27.77	4.497
7	*LOC613037*	2760189	351.17	533.33	4.401
8	*LRP3*	730458	457.68	191.43	4.397
9	*LOC729978*	6980601	2568.68	4299.57	4.389
10	*S100Z*	5870338	187.77	90.42	4.290
11	*FLJ40113*	2320603	-9.07	6.02	4.286
12	*LRP5L*	540403	141.42	276.77	4.269

Table S4. Differentially methylated probes between controls and unaffected offspring

Rank	Index	Target D	Controls AVG Beta	Offspring AVG Beta	Delta Beta	Illumina Gene Annotation
1	10788	cg05593887	0.17765	0.82772	0.65007	*MAGI2*
2	439894	cg25099095	0.71343	0.18537	-0.52806	
3	232939	cg12515659	0.20226	0.6958	0.49354	*FAM134B*
4	244024	cg13232075	0.26133	0.73814	0.4768	
5	164958	cg08522473	0.46799	0.92582	0.45783	*TAGLN3*
6	485539	rs2208123	0.63222	0.18306	-0.44916	
7	420903	cg23943944	0.73918	0.2918	-0.44738	
8	341341	cg18816122	0.10999	0.54603	0.43604	*PLEKHG4B*
9	418678	cg23804921	0.66943	0.23467	-0.43475	
10	125395	cg06417478	0.49136	0.06335	-0.42801	*HOOK2*
11	825	cg00033213	0.76162	0.34312	-0.4185	*TOP1MT*
12	140429	cg07179329	0.81228	0.40225	-0.41004	*CDH13*
13	485542	rs2468330	0.40563	0.80961	0.40398	
14	357637	cg19848924	0.8485	0.44559	-0.40292	
15	214900	cg11418607	0.20225	0.59598	0.39373	
16	485572	rs9292570	0.47504	0.8672	0.39216	
17	1113	cg00045070	0.2168	0.59676	0.37996	*PCSK9*
18	275290	cg14795227	0.66052	0.28297	-0.37755	
19	145092	cg07437919	0.52561	0.14916	-0.37645	*SLC45A4*
20	236587	cg12743416	0.43029	0.05617	-0.37412	*TRIM24*
21	178385	cg09289202	0.32759	0.70086	0.37327	*STK25*
22	151617	cg07791065	0.48947	0.85983	0.37036	
23	297138	cg16147201	0.70586	0.33572	-0.37014	
24	59125	cg02907150	0.42158	0.78546	0.36387	*PCNX*
25	27242	cg01303420	0.73203	0.37015	-0.36188	*C3orf55*
26	214578	cg11401796	0.84504	0.49041	-0.35463	*C21orf70*
27	162978	cg08412936	0.74738	0.40151	-0.34588	*PSORS1C1*
28	163134	cg08422420	0.12593	0.46952	0.34359	*SDHAP3*
29	190626	cg10000195	0.83652	0.49385	-0.34267	
30	220895	cg11792281	0.54053	0.19843	-0.3421	*NLK*
31	424315	cg24136292	0.19632	0.53542	0.33909	*INSC*
32	154968	cg07973095	0.21551	0.55264	0.33713	*DECR2*
33	461936	cg26472636	0.18363	0.52064	0.33701	*SDHAP3*
34	328253	cg18030105	0.6985	0.36216	-0.33634	*ZNF195*
35	157273	cg08104845	0.70519	0.36939	-0.3358	*GLUL*
36	288040	cg15600437	0.87072	0.53831	-0.3324	*MFAP2*
37	182703	cg09533869	0.12189	0.45421	0.33232	*PGCP*
38	169447	cg08778598	0.15171	0.4837	0.33199	*SDHAP3*
39	404358	cg22926869	0.6972	0.36743	-0.32977	*HHLA2*

40	300021	cg16316162	0.03761	0.36569	0.32808	*NAPRT1*
41	377036	cg21117559	0.66503	0.33811	-0.32692	*STARD13*
42	376323	cg21070081	0.51683	0.84299	0.32615	
43	223895	cg11957130	0.61246	0.28831	-0.32415	*ATXN7L1*
44	246480	cg13379757	0.78811	0.46513	-0.32298	
45	452582	cg25929399	0.19389	0.51681	0.32292	*KRT38*
46	192367	cg10098373	0.77258	0.45028	-0.32229	*CPT1A*
47	485554	rs5926356	0.35218	0.67312	0.32094	
48	479924	cg27521571	0.82631	0.50634	-0.31997	*COMT*
49	218975	cg11680857	0.69479	0.37499	-0.3198	*LCE2D*
50	302831	cg16471877	0.6782	0.35857	-0.31963	*COQ3*
51	151367	cg07777042	0.85598	0.53782	-0.31816	
52	282760	cg15251140	0.64789	0.33263	-0.31526	
53	420129	cg23899408	0.47816	0.16322	-0.31494	*HOOK2*
54	389523	cg21931717	0.08616	0.39962	0.31345	*SDHAP3*
55	52721	cg02587993	0.88235	0.56971	-0.31264	
56	321732	cg17605604	0.60023	0.28846	-0.31177	*C5orf30*
57	87981	cg04383836	0.64893	0.33766	-0.31127	*USP43*
58	28217	cg01356752	0.83381	0.52285	-0.31096	
59	237381	cg12798564	0.7643	0.45567	-0.30864	
60	338890	cg18669823	0.62958	0.32139	-0.30819	
61	430609	cg24534774	0.26134	0.56927	0.30793	
62	485521	rs10936224	0.64588	0.33836	-0.30751	
63	485534	rs1941955	0.69993	0.39409	-0.30584	
64	301482	cg16398051	0.77396	0.46897	-0.30499	*ADAMTS17*
65	437392	cg24937727	0.18563	0.49058	0.30495	*RGL3*
66	1158	cg00047185	0.76042	0.45612	-0.3043	*MIR526B*
67	167786	cg08683088	0.72006	0.41583	-0.30424	*ELOVL2*
68	355076	cg19707653	0.7376	0.4353	-0.3023	*KIAA1671*
69	74862	cg03706056	0.23804	0.54032	0.30228	*SETD4*
70	199608	cg10508111	0.8335	0.53137	-0.30212	*PFKM*
71	28700	cg01381374	0.60786	0.30602	-0.30184	
72	100886	cg05059349	0.24652	0.54792	0.3014	
73	108689	cg05477582	0.79757	0.49626	-0.30131	*CMTM1*
74	383609	cg21544585	0.80007	0.49928	-0.30079	*TMEM155; LOC100192379*
75	263104	cg14179288	0.5044	0.80462	0.30022	*TLE4*

Table S5. Differentially methylated probes between controls and BD patients

Rank	Index	Target ID	Controls AVG Beta	BD AVG Beta	Delta Beta	Illumina Gene Annotation
1	348332	cg19285525	0.20847	0.68924	0.48078	*RBMS1*
2	244024	cg13232075	0.26133	0.74139	0.48005	
3	448561	cg25649515	0.44069	0.91658	0.47589	
4	110788	cg05593887	0.17765	0.63184	0.45419	*MAGI2*
5	383107	cg21514997	0.15867	0.5992	0.44054	*CCDC148*
6	125395	cg06417478	0.49136	0.05731	-0.43405	*HOOK2*
7	182703	cg09533869	0.12189	0.54351	0.42162	*PGCP*
8	485542	rs2468330	0.40563	0.80799	0.40235	
9	485561	rs6546473	0.40975	0.8096	0.39985	
10	485566	rs739259	0.22973	0.62793	0.3982	
11	389458	cg21927991	0.85012	0.457	-0.39312	*ZFAT*
12	261966	cg14123034	0.57669	0.18501	-0.39169	*L3MBTL4*
13	485538	rs213028	0.33747	0.72259	0.38512	
14	116088	cg05890457	0.45391	0.83665	0.38274	*ARHGEF10*
15	246480	cg13379757	0.78811	0.40578	-0.38234	
16	485516	rs10457834	0.72943	0.35151	-0.37792	
17	447746	cg25599641	0.92394	0.55674	-0.3672	*NAGA*
18	311144	cg16954525	0.32197	0.68911	0.36713	*MS4A4A*
19	145092	cg07437919	0.52561	0.15922	-0.36639	*SLC45A4*
20	59125	cg02907150	0.42158	0.78787	0.36629	*PCNX*
21	223881	cg11956442	0.41016	0.77368	0.36352	
22	365865	cg20381372	0.33843	0.70088	0.36245	*LOC100134317*
23	367053	cg20459037	0.68591	0.32398	-0.36193	*WDR16*
24	157273	cg08104845	0.70519	0.34573	-0.35946	*GLUL*
25	161458	cg08320316	0.43367	0.78756	0.35389	*RNF216*
26	119400	cg06064179	0.79699	0.44637	-0.35062	*PLEKHM1P*
27	418678	cg23804921	0.66943	0.31977	-0.34966	
28	472472	cg27076160	0.74071	0.39342	-0.34729	*ZNF365*
29	213402	cg11331837	0.42954	0.7728	0.34326	
30	407120	cg23084506	0.54129	0.20045	-0.34084	*PRAMEF5*
31	220895	cg11792281	0.54053	0.20127	-0.33926	*NLK*
32	170144	cg08820231	0.30405	0.64127	0.33723	*SLC26A10*
33	7278	cg00345083	0.30289	0.63962	0.33674	*AJAP1*
34	394844	cg22304519	0.73788	0.40221	-0.33567	
35	485531	rs1510189	0.3942	0.72938	0.33518	
36	420129	cg23899408	0.47816	0.14788	-0.33028	*HOOK2*
37	294557	cg15991478	0.81759	0.48735	-0.33024	*UBD*
38	357637	cg19848924	0.8485	0.51997	-0.32854	
39	96767	cg04850148	0.37416	0.70098	0.32682	*CCL4L2; CCL4L1*

40	146302	cg07501029	0.39536	0.72178	0.32643	*KIF26B*
41	251419	cg13612055	0.49681	0.8211	0.3243	*RNU5E; RNU5D; ACOT12*
42	140933	cg07205452	0.90026	0.57751	-0.32274	*PRDM10*
43	374956	cg20979384	0.28228	0.60258	0.3203	
44	143037	cg07319199	0.83807	0.51884	-0.31923	*ANTXR1*
45	200687	cg10568066	0.3007	0.61986	0.31917	*RNF39*
46	190626	cg10000195	0.83652	0.51888	-0.31764	
47	207438	cg10975354	0.1159	0.43351	0.31761	*VPS13A; LOC100286938*
48	478174	cg27415324	0.72948	0.41529	-0.31419	
49	36864	cg01800061	0.91661	0.60309	-0.31352	*MIR519B; MIR526B*
50	125277	cg06409538	0.54335	0.23001	-0.31334	
51	328253	cg18030105	0.6985	0.38556	-0.31294	*ZNF195*
52	468599	cg26856631	0.50776	0.19611	-0.31164	*LSM5*
53	485565	rs715359	0.66521	0.35434	-0.31088	
54	189001	cg09900440	0.75651	0.44819	-0.30832	*HLA-DPA1*
55	323047	cg17707870	0.12268	0.43009	0.3074	*LHFP*
56	303705	cg16523115	0.59026	0.89605	0.30578	*IL20RB*
57	116629	cg05918715	0.77954	0.47426	-0.30528	*SHISA2*
58	218975	cg11680857	0.69479	0.38959	-0.3052	*LCE2D*
59	275290	cg14795227	0.66052	0.35658	-0.30394	
60	27242	cg01303420	0.73203	0.42815	-0.30389	*C3orf55*
61	485575	rs951295	0.36173	0.66553	0.30379	
62	302713	cg16464924	0.56818	0.87158	0.30339	*GAA*
63	461459	cg26445985	0.34149	0.64327	0.30177	*DENND2A*
64	166866	cg08629394	0.41257	0.71276	0.30018	

Table S6. Differentially methylated probes between unaffected offspring and bipolar disorder patients

Rank	Index	Target ID	Offspring AVG Beta	BD AVG Beta	Delta Beta	Illumina Gene Annotation
1	261966	cg14123034	0.69639	0.18379	-0.5126	*L3MBTL4*
2	232939	cg12515659	0.68621	0.21166	-0.47455	*FAM134B*
3	485539	rs2208123	0.18016	0.6173	0.43714	
4	1113	cg00045070	0.5902	0.16964	-0.42056	*PCSK9*
5	162978	cg08412936	0.39844	0.81823	0.41979	*PSORS1C1*
6	420903	cg23943944	0.29044	0.70992	0.41947	
7	145228	cg07446795	0.87965	0.46651	-0.41313	*CCDC40*
8	140429	cg07179329	0.40153	0.8042	0.40267	*CDH13*
9	116300	cg05900567	0.59664	0.19687	-0.39976	
10	485538	rs213028	0.3321	0.72101	0.38891	
11	460690	cg26398228	0.34913	0.72761	0.37848	
12	407120	cg23084506	0.56815	0.19767	-0.37048	*PRAMEF5*
13	119400	cg06064179	0.79083	0.42867	-0.36217	*PLEKHM1P*
14	485537	rs2125573	0.73229	0.37576	-0.35653	
15	6860	cg00325917	0.48153	0.8371	0.35558	*SELL*
16	164958	cg08522473	0.92101	0.57185	-0.34915	*TAGLN3*
17	179474	cg09351263	0.54725	0.19839	-0.34886	
18	419138	cg23836570	0.5326	0.87885	0.34625	*FBRSL1*
19	213127	cg11314779	0.8831	0.53801	-0.34509	
20	485518	rs10796216	0.55979	0.21687	-0.34292	
21	396292	cg22402398	0.74372	0.4018	-0.34192	*FGR*
22	350115	cg19405842	0.88288	0.54172	-0.34116	*PRKCZ*
23	223881	cg11956442	0.42945	0.76728	0.33783	
24	463816	cg26576978	0.45006	0.78547	0.33541	
25	116108	cg05891136	0.58946	0.92431	0.33486	*MYOM2*
26	476971	cg27341708	0.24295	0.57466	0.33171	*MAML3*
27	202953	cg10701801	0.06483	0.39634	0.33151	*OSBPL9*
28	189001	cg09900440	0.77725	0.44612	-0.33113	*HLA-DPA1*
29	178385	cg09289202	0.69895	0.37018	-0.32877	*STK25*
30	153179	cg07876831	0.76254	0.43471	-0.32783	*TMCO3*
31	301482	cg16398051	0.46819	0.79593	0.32774	*ADAMTS17*
32	66803	cg03299990	0.32387	0.6491	0.32523	
33	235770	cg12687426	0.59724	0.92012	0.32288	*KCNMB3*
34	74862	cg03706056	0.5352	0.21428	-0.32092	*SETD4*
35	348332	cg19285525	0.36288	0.68247	0.3196	*RBMS1*
36	151367	cg07777042	0.52756	0.84707	0.3195	
37	687	cg00028022	0.5443	0.22536	-0.31894	
38	375699	cg21028319	0.67159	0.35529	-0.3163	*TM2D2*

39	311264	cg16963093	0.69048	0.37654	-0.31394	*ATXN7L1*
40	297138	cg16147201	0.33515	0.64603	0.31089	
41	21693	cg01029331	0.61995	0.9284	0.30845	*SDHAP1*
42	485516	rs10457834	0.65718	0.34991	-0.30728	
43	439894	cg25099095	0.1846	0.49178	0.30718	
44	185098	cg09672255	0.53597	0.84182	0.30585	*ZNF879*
45	485572	rs9292570	0.86523	0.56142	-0.30382	
46	36864	cg01800061	0.89669	0.59348	-0.30321	*MIR519B; MIR526B*
47	395319	cg22337626	0.66367	0.361	-0.30266	*MAST2*

Table S7. Top networks enriched in the list of risk genes for BD

ID	Molecules in Network	Score	Focus Molecules	Top Diseases and Functions
1	2010109I03Rik, ADRB2, **ALDH6A1**, APP, **ARID4B**, **ATP6V1F**, beta-estradiol, C14orf93, CASP3, **CLASRP**, **CLPTM1**, **COG5**, FOS, **GLUL**, GPR61, GPR78, GPR85, GPR88, GPR139, GPR173, **HINT3**, **HOOK2**, IGSF6, **LSM14B**, **MAGI2**, MOV10, NTRK1, **NUCKS1**, **PBX2**, **PCNX**, **PEX2**, PIK3R2, **PRR14**, PTTG2, ZNF343	37	16	Cancer, Organismal Injury and Abnormalities, Reproductive System Disease
2	Androgen-AR, CACHD1, CD3, CDC42, CDC42SE1, CDC42SE2, CYP1A1, DNAJB14, **DNAJC7**, DNAJC8, EML4, **FBXW11**, **GTF2A1**, **HSPA1L**, **HUWE1**, **IL7R**, indonel, INF2, LPIN2, MAP2K4/7, **MED1**, **MTF2**, NFkB (complex), POLR2F, POLR2K, RNA pol2-transcription factor, **SAFB2**, **SNORA65**, SUMO4, **SUZ12**, **TAF15**, **THOC2**, TRIM52, ZKSCAN1, **ZNF234**	32	14	Cellular Development, Hematological System Development and Function, Hematopoiesis
3	Anti-inflammatory Cytokine, **ATP8B2**, ATP9A, CHST3, **CPQ**, **FASTK**, GATM, GCC2, GNA14, IL4, ISG15, ITGBL1, ITPA, KITLG, **LCE2C/LCE2D**, LOXL1, LRRC32, METRNL, MNK1/2, MPHOSPH9, MYB, **NLK**, **PITPNM1**, RNF152, PNF220, SCGB3A1, SPRY1, TFAP2A, TGFB1, Tnfrsf22/Tnfrsr23, **UBE2E1**, VTCN1, Zfp35, **ZNF195**, **ZNF641**	19	9	Hematological System Development and Function, Tissue Morphology, Cellular Development
4	LHX1, **SLC45A4**	3	1	Cell Death and Survival, Cellular Movement, Nervous System Development and Function

Table S8. List of genes showing a very strong correlation between expression and methylation (r^2> | 0.8 |).

Rank	Gene	Pearson's R
1	*HLA-DRB5*	0.9832
2	*HLA-DRB5*	0.97487
3	*HLA-DRB5*	0.95908
4	*RAB34*	-0.94641
5	*RAB34*	-0.94073
6	*RAB34*	-0.94067
7	*HLA-DRB5*	0.93113
8	*PBX2*	-0.92892
9	*KIFC1*	-0.92295
10	*ZNF384*	0.9093
11	*RAB34*	-0.9045
12	*RIMBP2*	-0.89906
13	*NUCKS1*	0.89758
14	*HLA-DRB5*	0.89693
15	*RAB34*	-0.89405
16	*RAB34*	-0.89279
17	*UBAC2*	-0.87961
18	*TADA3*	0.87778
19	*RAB34*	-0.87489
20	*SUPT6H*	-0.87316
21	*PBX2*	-0.86884
22	*CENPT*	0.86767
23	*HLA-DRB5*	0.86722
24	*PGAM4*	0.86576
25	*HSPA1L*	0.86424
26	*PBX2*	-0.86097
27	*RAB43*	-0.86053
28	*NUCKS1*	0.86032
29	*MAP7*	-0.857
30	*MTF2*	0.85641
31	*HLA-DRB5*	0.85522
32	*FASTK*	0.85387
33	*EIF1AY*	-0.85039

Rank	Gene	Pearson's R
34	*MALT1*	0.84992
35	*SFRS18*	0.84987
36	*ANUBL1*	-0.84985
37	*PTPRN2*	-0.84947
38	*PTPRN2*	-0.84947
39	*PSMB8*	0.84916
40	*ADARB2*	-0.84903
41	*GABRG2*	-0.84892
42	*TFDP1*	-0.84777
43	*PRX*	0.84697
44	*FAM13A*	-0.84664
45	*HSPA1L*	0.84606
46	*QKI*	-0.84454
47	*E2F4*	0.8439
48	*ASF1B*	0.84344
49	*MBNL3*	0.84257
50	*ADPGK*	-0.8411
51	*KDM2B*	0.84085
52	*TAF1C*	0.84057
53	*PTPRN2*	-0.83875
54	*ZNF692*	0.83808
55	*BZW2*	0.83703
56	*BCL2*	-0.83665
57	*SFRS18*	0.83653
58	*SEPT9*	-0.83409
59	*LSM14B*	-0.83365
60	*NUCKS1*	-0.83333
61	*METRNL*	0.83154
62	*USP5*	0.83114
63	*EIF1AY*	-0.83085
64	*CD44*	-0.83077
65	*MAML3*	0.83076
66	*MRAS*	-0.82975

Rank	Gene	Pearson's R
67	*TIAF1*	-0.82758
68	*DDX46*	0.82721
69	*LDHB*	0.82517
70	*HLA-DRB5*	-0.82501
71	*KIAA1737*	-0.82487
72	*MYOM1*	0.82189
73	*ZNF384*	0.82185
74	*SAE1*	0.8196
75	*PTPRN2*	-0.81954
76	*CPSF1*	0.81819
77	*NUCKS1*	0.818
78	*ENC1*	0.81718
79	*SLC22A17*	0.81707
80	*CCR4*	0.81637
81	*PRDM16*	-0.81578
82	*DCUN1D4*	-0.81524
83	*MCM6*	0.81513
84	*ITPKB*	-0.81471
85	*SMAD3*	-0.81456
86	*PTTG1*	-0.81433
87	*CSMD1*	-0.81405
88	*PGAM4*	0.8137
89	*KIF17*	-0.81316
90	*RPS6KA3*	-0.81309
91	*PUM1*	0.81179
92	*TIGD1*	0.81148
93	*ZNF92*	-0.81147
94	*SEPT9*	0.81115
95	*RAB11B*	-0.81079
96	*CD248*	-0.8105
97	*PBX2*	-0.80972
98	*EDN3*	-0.80961
99	*ERMN*	-0.80952
100	*EN1*	0.80884

Rank	Gene	Pearson's R
101	*KIAA1530*	0.80869
102	*PCNA*	-0.80854
103	*HOXC4;HOXC6;HOXC5*	0.80815
104	*C19orf6*	0.80808
105	*CTDP1*	-0.80797
106	*MAP7*	-0.80708
107	*RNF212*	-0.80683
108	*UGP2*	0.80669
109	*BAT2*	0.80626
110	*FTH1*	-0.80615
111	*NSUN5*	-0.80603
112	*ACTN1*	-0.80561
113	*RWDD1*	0.80555
114	*ATP5D*	0.80539
115	*MAP4K4*	0.80475
116	*MGST1*	-0.80432
117	*NUCKS1*	0.8043
118	*ADPRHL1*	-0.80421
119	*ATP11A*	-0.80337
120	*IQCK*	0.80307
121	*SEC14L1*	-0.80244
122	*KIAA1407*	0.80221
123	*FAM98A*	-0.80198
124	*HNRNPUL2;TTC9C*	0.8019
125	*SCRIB*	-0.80156
126	*SLC29A1*	-0.80153
127	*GSTTP2*	-0.80141
128	*HNRNPA0*	-0.80124
129	*FBXL19*	-0.80119
130	*PHF5A;ACO2*	0.801
131	*CENPT*	0.80095
132	*HSPB1*	-0.80068
133	*C14orf119;ACIN1*	0.80065
134	*USF2*	0.80037
135	*TAF15*	0.80004

References

1. Andersen CL, Jensen JL, Orntoft TF. Normalization of real-time quantitative reverse transcription-PCR data: a model-based variance estimation approach to identify genes suited for normalization, applied to bladder and colon cancer data sets. *Cancer Res* 2004; **64**:5245-50.
2. Livak KJ, Schmittgen TD. Analysis of relative gene expression data using real-time quantitative PCR and the 2(-Delta Delta C(T)) Method. *Methods* 2001; **25**:402-8.
3. Tryka KA, Hao L, Sturcke A, Jin Y, Wang ZY, Ziyabari L, et al. NCBI's Database of Genotypes and Phenotypes: dbGaP. *Nucleic Acids Res* 2014; **42**:D975-9.
4. Cichon S, Schumacher J, Muller DJ, Hurter M, Windemuth C, Strauch K, et al. A genome screen for genes predisposing to bipolar affective disorder detects a new susceptibility locus on 8q. *Hum Mol Genet* 2001; **10**:2933-44.
5. Gonzalez S, Camarillo C, Rodriguez M, Ramirez M, Zavala J, Armas R, et al. A genome-wide linkage scan of bipolar disorder in Latino families identifies susceptibility loci at 8q24 and 14q32. *Am J Med Genet B Neuropsychiatr Genet* 2014; **165b**:479-91.
6. Lekman M, Karlsson R, Graae L, Hossjer O, Kockum I. A significant risk locus on 19q13 for bipolar disorder identified using a combined genome-wide linkage and copy number variation analysis. *BioData Min* 2015; **8**:42.
7. Francks C, Tozzi F, Farmer A, Vincent JB, Rujescu D, St Clair D, et al. Population-based linkage analysis of schizophrenia and bipolar case-control cohorts identifies a potential susceptibility locus on 19q13. *Mol Psychiatry* 2010; **15**:319-25.
8. Georgi B, Craig D, Kember RL, Liu W, Lindquist I, Nasser S, et al. Genomic view of bipolar disorder revealed by whole genome sequencing in a genetic isolate. *PLoS Genet* 2014; **10**:e1004229.
9. Badner JA, Gershon ES. Meta-analysis of whole-genome linkage scans of bipolar disorder and schizophrenia. *Mol Psychiatry* 2002; **7**:405-11.

10. Large-scale genome-wide association analysis of bipolar disorder identifies a new susceptibility locus near ODZ4. *Nat Genet* 2011; **43**:977-83.

11. Clelland CL, Read LL, Panek LJ, Nadrich RH, Bancroft C, Clelland JD. Utilization of never-medicated bipolar disorder patients towards development and validation of a peripheral biomarker profile. *PLoS One* 2013; **8**:e69082.

12. Savitz J, Frank MB, Victor T, Bebak M, Marino JH, Bellgowan PS, et al. Inflammation and neurological disease-related genes are differentially expressed in depressed patients with mood disorders and correlate with morphometric and functional imaging abnormalities. *Brain Behav Immun* 2013; **31**:161-71.
