# Supplementary figures and images for: Integrated transcriptome and methylome analysis in youth at high risk for bipolar disorder: a preliminary analysis
Source: Transl Psychiatry. 2017 Mar 14;7(3):e1059–. doi: 10.1038/tp.2017.32 (PMC5416675; doi:10.1038/tp.2017.32)

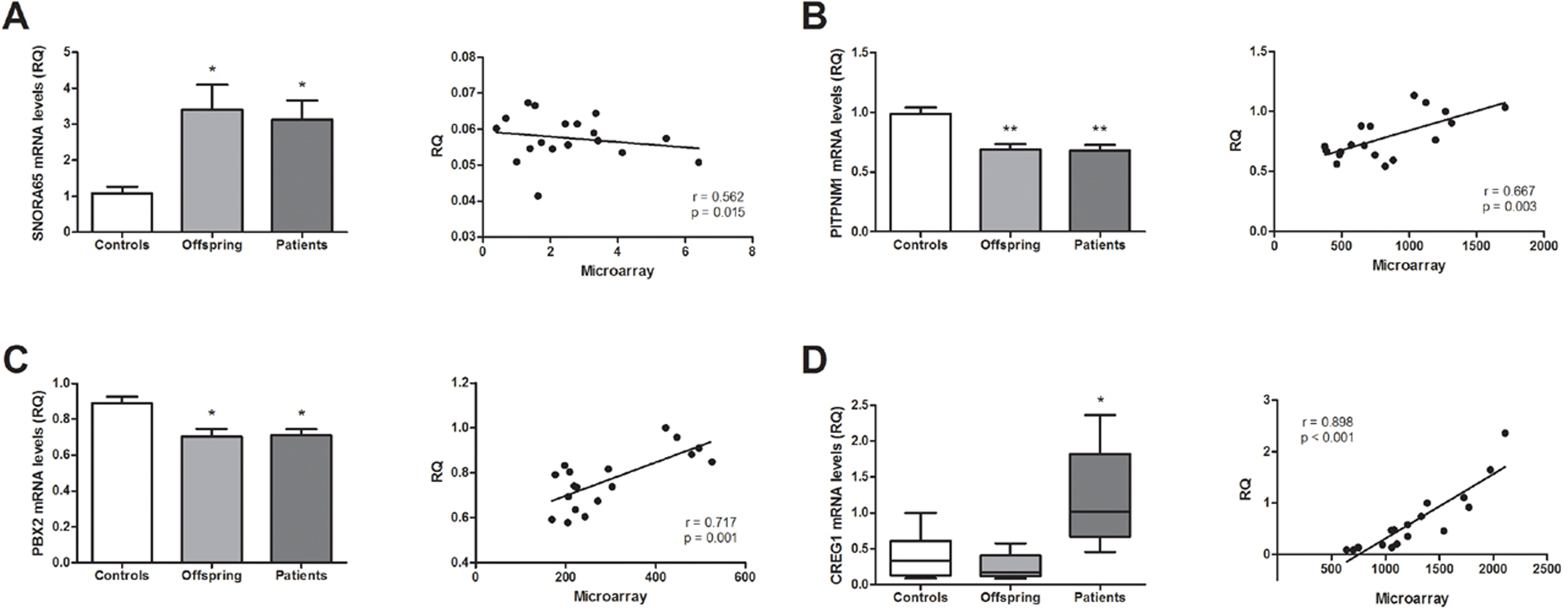

Supplement: Supplementary Figure 1 [file tp201732x2.tif]

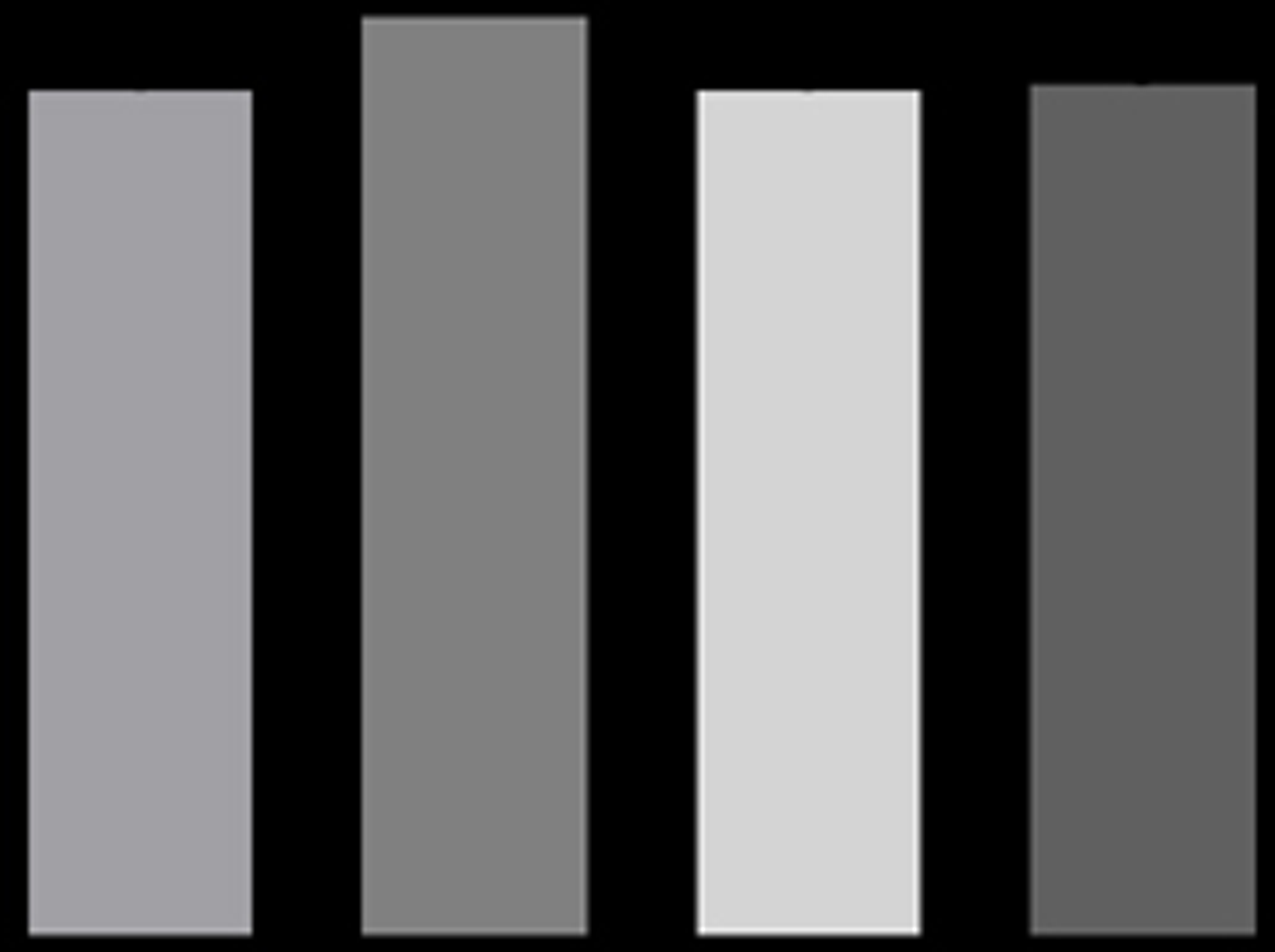

Supplement: Supplementary Figure 2 [file tp201732x3.tif]

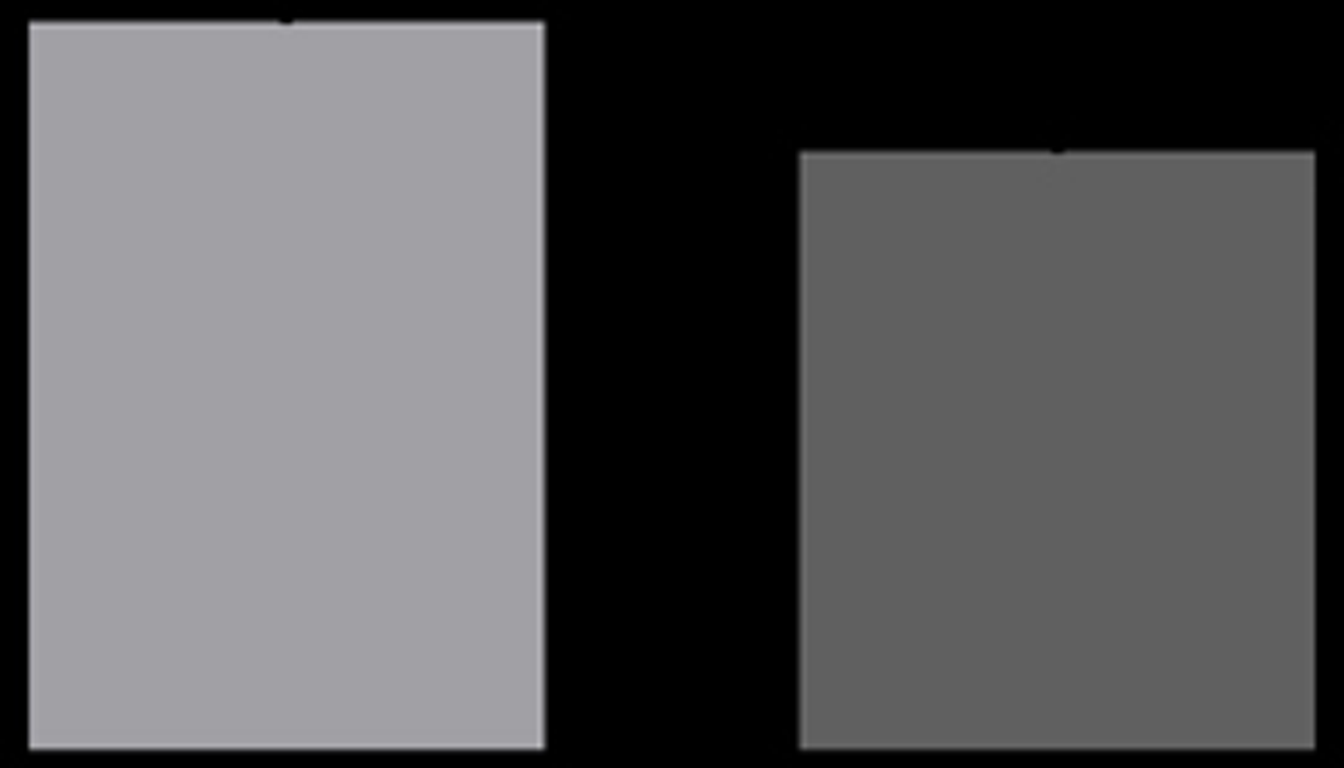

Supplement: Supplementary Figure 3 [file tp201732x4.tif]
